# Acuity and summation strategies differ in vinegar and desert fruit flies

**DOI:** 10.1016/j.isci.2021.103637

**Published:** 2021-12-16

**Authors:** John P. Currea, Rachel Frazer, Sara M. Wasserman, Jamie Theobald

**Affiliations:** 1 Department of Psychology, Florida International University, Miami, FL 33199, USA; 2 Division of Neurobiology and Behavior, Columbia University, New York, NY 10027, USA; 3 Department of Neuroscience, Wellesley College, Wellesley, MA 02481, USA; 4 Department of Biological Sciences, Florida International University, Miami, FL 33199, USA

**Keywords:** Biological sciences, Neuroscience, Neuroanatomy, Sensory neuroscience

## Abstract

An animal's vision depends on terrain features that limit the amount and distribution of available light. Approximately 10,000 years ago, vinegar flies (*Drosophila melanogaster*) transitioned from a single plant specialist into a cosmopolitan generalist. Much earlier, desert flies (*D. mojavensis*) colonized the New World, specializing on rotting cactuses in southwest North America. Their desert habitats are characteristically flat, bright, and barren, implying environmental differences in light availability. Here, we demonstrate differences in eye morphology and visual motion perception under three ambient light levels. Reducing ambient light from 35 to 18 cd/m^2^ causes sensitivity loss in desert but not vinegar flies. However, at 3 cd/m^2^, desert flies sacrifice spatial and temporal acuity more severely than vinegar flies to maintain contrast sensitivity. These visual differences help vinegar flies navigate under variably lit habitats around the world and desert flies brave the harsh desert while accommodating their crepuscular lifestyle.

## Highlights

• Fruit fly species from stark deserts have larger, wider eyes than Vinegar flies
• Desert flies have more facets and, in bright light, higher spatial acuity
• As light dims, Vinegar flies lose spatial and temporal acuity to retain sensitivity
• Desert flies, conversely, retain more acuity at the cost of sensitivity

## Graphical abstract

**Figure:**
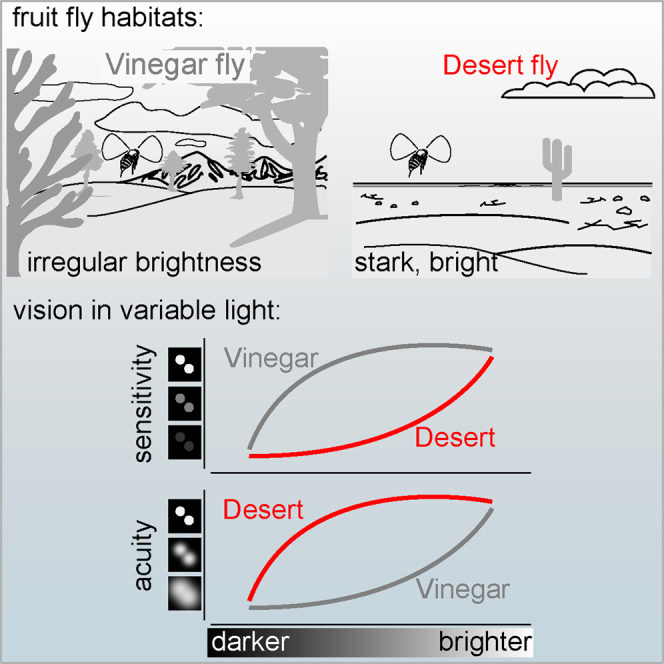

## Introduction

**Figure 1:**
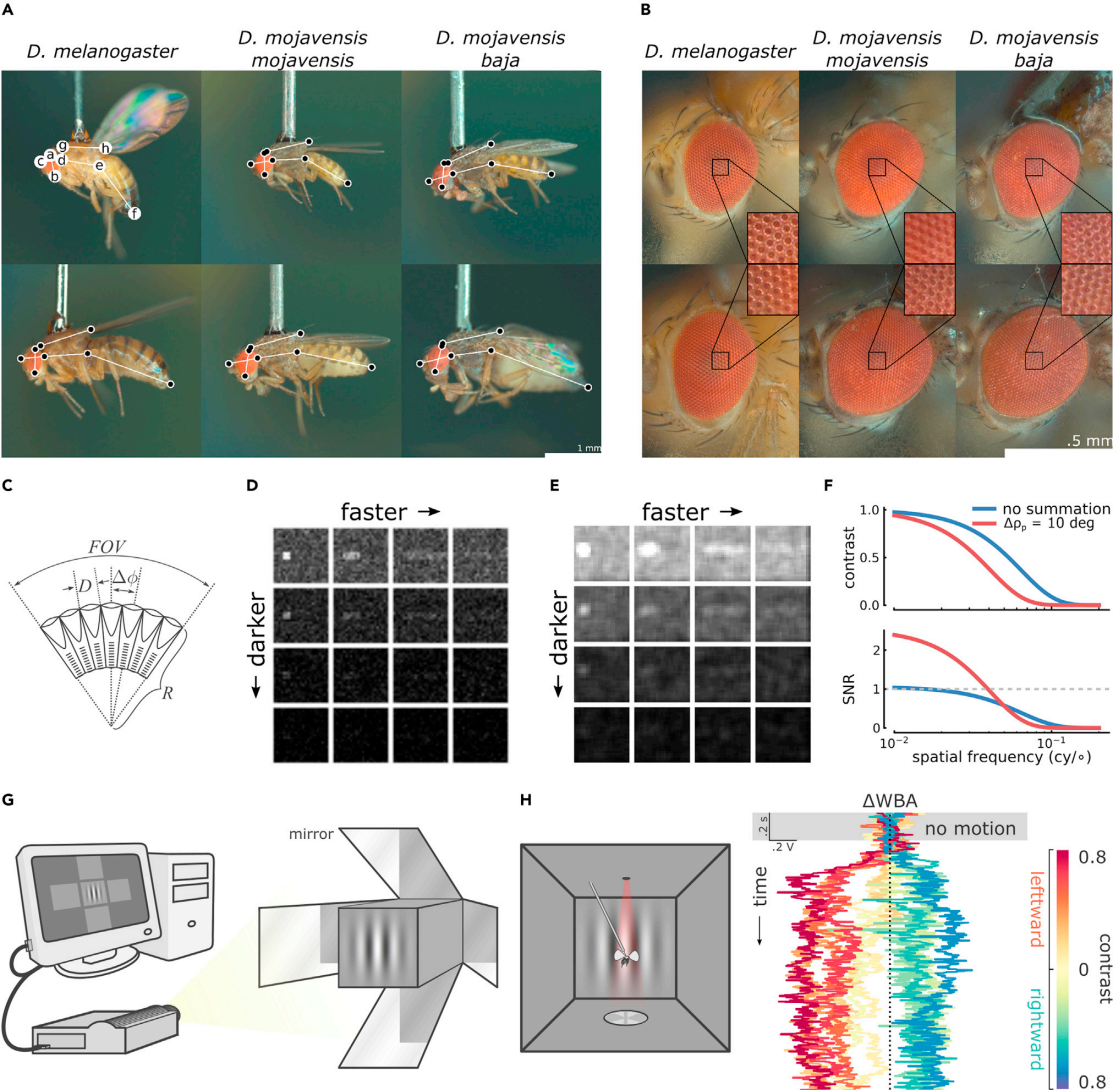
Our methodology included a combination of microscopy, allometry, optical modeling, and virtual reality flight simulation (A) One small (top row) and large (bottom row) subject from each species is displayed in profile view. The letters or black spots within white circles indicate landmarks used in measuring how abdomen ($\bar{ef}$), thorax ($\bar{gh}$), head ($\bar{cd}$), and eye lengths ($\bar{ab}$*)* scale with respect to body length ($\bar{cdef}$). Scale bar is 1 mm. (B) Likewise, one small and large eye from each species is displayed to give a sense of their eye allometry, with each inset zooming in to resolve the individual ommatidia on the eye surface. Scale bar is 0.5 mm. (C) Contrast sensitivity is partially limited by *D*, the facet diameter, and spatial acuity is inversely limited by *Δφ*, the interommatidial angle. In a spherical eye, *Δφ* = *D*/*R*, and the field of view, *FOV*, is equal to the sum of interommatidial angles subtended. (D) Owing to shot noise, lower ambient light levels or higher image speeds reduce image contrast. (E) Spatial summation of the images in panel (D) sacrifices spatial detail in order to improve visibility, even when ambient light levels decrease or image speed increases. (F) Models for the *Drosophila* eye modulation transfer function and signal-to-noise ratio (SNR) per spatial frequency with and without spatial summation. Despite reducing overall Michelson contrast of the image (top), spatial summation increases the signal-to-noise ratio of lower spatial frequencies (bottom). (G) A computer generates sinusoidal moving gratings projected at 360 Hz onto 5 sides of the perspex cube using 4 angled mirrors. (H) In the flight arena, a shadow of both wings is cast by an IR LED and their wingbeats are recorded independently by two photodiodes at 1 kHz (left). The difference in left and right wingbeat amplitudes (ΔWBA) is proportional to yaw torque and implies their perception of motion via the optomotor response (right). The ΔWBA time series collected from one subject in response to 11 different grating conditions are plotted, with colors indicating grating conditions: a grating of .0 contrast showing no motion (yellow) and 5 contrasts (.09, .28, .47, .66, and .85) moving toward the left (warm colors) and right (cool colors).

The vinegar fly (*Drosophila melanogaster*; Figure 1A, left column) buzzing around your ripe fruit or open beer, is surprisingly widespread (Markow, 2015). Once mostly restricted to sub-Saharan Africa (Throckmorton, 1975) where they used rotting fruit, likely marula, as their host (Karageorgi et al., 2018; Mansourian et al., 2018; Markow, 2015), they dispersed to Asia and Europe around 10 thousand years ago, and then with humans to Australia and the Americas within the past few centuries (David and Capy, 1988; Markow, 2015). As a human commensal, vinegar flies transitioned to a cosmopolitan generalist lifestyle, targeting fruits around the world (Atkinson and Shorrocks, 1977; Karageorgi et al., 2018; Mansourian et al., 2018). However, flies from the *Drosophila* (*Siphlodora*) subgenus (Yassin, 2013) beat vinegar flies to the New World by about 30 million years, radiating first in South America (O'Grady and DeSalle, 2018; Smith et al., 2012; Throckmorton, 1975). Members of what would become the *repleta* species group shifted to feeding and breeding on fermenting cactuses, likely from arid regions of Peru and Bolivia (Smith et al., 2012), and many followed cactus populations dispersing northward. One cactophilic fly (*D. mojavensis*, Figure 1A, middle and right columns) colonized deserts of southwest North America, diverging from Mexico over the past ∼300,000 years into subspecies specializing on various endemic cactuses (Mojave, Sonoran, and Baja California; Smith et al., 2012). Because 1) their ecologies are unique and include a history of host shifts, geographic isolation, chromosomal inversions, and incipient speciation, 2) the *repleta* group is monophyletic, and, 3) among other cactophilic species, the desert fly has had its genome sequenced and shared publicly (Drosophila 12 Genomes Consortium, 2007), cactophilic flies are a model system for studying genetics, ecology, speciation, and their interactions (Markow, 2019; Smith et al., 2012).

Although their eyes are superficially similar, profound ecological differences have driven vinegar and desert flies to see differently. Desert flies have larger eyes absolutely (Figure 1B), and relative to their antennae (Keesey et al., 2019). Vinegar flies correct for visual sideslip above and below their head, but desert flies suppress responses to all sideslip above and some below, responding specifically to the low visual clutter expected in barren deserts (Ruiz, 2021). Vinegar flies also steer away from small visual spots (Maimon et al., 2008; Park and Wasserman, 2018), which may help avoid predators (Maimon et al., 2008), but this response is absent in desert flies (Park and Wasserman, 2018). However, little is known about the general visual capacity of desert flies. Given that visual ecologies and responses both differ, what visual traits adapt desert flies to the unique desert landscape, in contrast to the cosmopolitan vinegar fly?

Species of the genus *Drosophila* wield neural superposition eyes of about 1,000 facets each (Figure 1B, insets; Keesey et al., 2019). Each facet, called an ommatidium, isolates light from neighbors by pigment cells, and directs light onto photoreceptors with a lens and crystalline cone (Ready et al., 1976). Lens size limits light collection over a fixed duration, called optical sensitivity, and thus, partially, the ability to differentiate brightness levels called contrast sensitivity, which is critical when under dim light or during fast image motion (Snyder et al., 1977a,1977b; Theobald, 2017; Warrant, 1999). The angular separation of ommatidia, called the interommatidial angle, limits the smallest discernible details and thus inversely limits resolution of spatial detail, called spatial acuity. Spatial acuity is necessary for object recognition, collision avoidance, and perceiving small changes in self-motion (Land, 1997; Land and Nilsson, 2012).

*Drosophila* eyes are approximately spherical, having a single radius of curvature across the eye and ommatidial axes converging to one approximate point (Stavenga, 1975, 1979). As a result, the interommatidial angle is approximated as *Δφ* = *D/R*, where *Δφ* is the interommatidial angle, *D* the facet diameter, and *R* the eye radius (Figure 1C; Land, 1997; Stavenga, 1979). The total visual angle defines the field of view (*FOV* in Figure 1C), the sum of interommatidial angles subtended. This geometric relation demonstrates a special case of how spatial acuity and optical sensitivity trade off (Land and Nilsson, 2012; Snyder et al., 1977a): for a given *R* and *FOV*, increasing sensitivity by increasing *D* decreases spatial acuity by decreasing 1/*Δφ*, and vice versa. Likewise, if *FOV* remains constant and *R* decreases, the eye must sacrifice 1) spatial acuity by decreasing 1/*Δφ* and consequently the number of ommatidia, 2) optical sensitivity by decreasing *D*, or 3) both. It is therefore illuminating to determine how parameters change with eye size, called allometry (Shingleton et al., 2007; Voje et al., 2014), such as in small vinegar flies that sacrifice optical sensitivity more than spatial acuity (Currea et al., 2018). Should the same hold for desert flies?

Deserts are often flat, open-country habitats where predators or cactuses adhere to a planar surface. Inhabitants of flat environments frequently possess wide FOVs and enhanced spatial acuity along the horizontal meridian (the terrain theory, Hughes, 1977), found among mammals, birds, reptiles, amphibians, and fish (Collin and Pettigrew, 1988; Johnson and Gadow, 1901; Luck, 1965; Potier et al., 2020; Pumphrey, 1948; Wood, 1917; for examples, see Hughes, 1977), and fiddler crabs (Brodrick et al., 2020; Smolka and Hemmi, 2009) backswimmers (*Notonecta*), water striders (*Gerridae*), and some empidid flies (Zeil et al., 1989). These are likely adaptations to horizons with few interruptions, not just open-country (Hughes, 1977), and seem absent in nocturnal open-country dwellers (Hughes, 1977; Lisney et al., 2012). And although the Saharan *Cataglyphis* ants have a horizontal streak of increased spatial acuity (Wehner et al., 2014), Australian desert ants (*Melophorus bagoti)* have a streak of increased optical sensitivity and *reduced* acuity (Schwarz et al., 2011), reflecting differences in lifestyle or clutter typical to each desert (Wehner et al., 2014). Evolution of the wide FOV and horizontal streak therefore depends on a visible horizon largely uninterrupted by vegetation or darkness, and the nature of the streak may depend on lifestyle or habitat image statistics.

Deserts often lack shade or terrain relief, and so provide unique tradeoffs for optical sensitivity and spatial acuity (Land, 1997; Snyder et al., 1977a, 1977b). *D* and *Δφ* (Figure 1C) are limited by diffraction through ommatidia, and the random nature of light absorption (Snyder et al., 1977b), and maximizing spatial information requires an eye at the diffraction limit, with a maximum discernible wavelength of *λ*_*max*_< 2*DΔφ* (Howard and Snyder, 1983; Snyder, 1979). Without a canopy, deserts are generally bright (Schwegmann et al., 2014), likely favoring smaller facet diameters (lower optical sensitivity) and narrower *Δφ* (higher spatial acuity), with *λ*_*max*_ closer to the upper limit of spectral sensitivity. Thus, small conspecifics may sacrifice contrast sensitivity to maintain competitive levels of horizontal FOV and spatial acuity.

Desert flies may therefore differ along parameters of eye morphology including corneal lens area, interommatidial angle, and FOV. However, vision is only partially determined by eye morphology due to neural processing of the visual system. Fortunately, flies and many seeing animals move their eyes and whole body in response to wide-field motion, called the optomotor response (Land, 2019; Land and Nilsson, 2012; Reichardt, 1962). The optomotor response offers a behavioral probe into the spatiotemporal performance of visual processing in both species by comparing the range of stimuli each species can respond to. Therefore, we can use the optomotor response to better understand the general visual differences between desert and vinegar flies and the behavioral consequences of morphological differences.

In particular, both desert and vinegar flies are active at sunrise and sunset (Hardeland and Stange, 1973). As light levels drop or image speed increases, the signal-to-noise ratio (SNR) of light absorption drops quadratically, reducing contrast sensitivity and the ability to resolve an image, such as in Figure 1D. To improve contrast sensitivity in dim light, animals can neurally summate over time or angular space (Snyder et al., 1977b; Warrant, 1999). Temporal summation collects photons for longer durations, similar to a long photographic exposure, improving contrast sensitivity at the cost of high temporal resolution (Warrant, 1999). Vinegar flies temporally summate in dim light (Juusola and Hardie, 2001; Palavalli-Nettimi and Theobald, 2020) and smaller flies temporally summate to achieve the contrast sensitivity of larger ones (Currea et al., 2018). Spatial summation increases the effective interommatidial angle, similar to using grainy photographic film, improving contrast sensitivity at the cost of high spatial resolution (Snyder, 1979; Warrant, 1999). For instance, spatial summation of the images in Figure 1D results in reduced spatial detail but increased visibility in the images of Figure 1E. This effect is elucidated by comparing models for the modulation transfer function and SNR of the *Drosophila* eye with and without spatial summation (using equations from Warrant, 1999). Visibility improves because, despite reducing overall contrast (Figure 1F, top), summation increases the SNR of lower spatial frequencies (Figure 1F, bottom). Spatial summation is a common response to low light or fast image velocity (Theobald et al., 2010; Warrant, 2017) likely implemented in flies by laterally extending dendrites of lamina monopolar cells or electrical coupling of photoreceptors within an ommatidium (Dubs et al., 1981; Greiner et al., 2005; Stöckl et al., 2020; Warrant et al., 2004). Vinegar flies spatially summate in the dark (Palavalli-Nettimi and Theobald, 2020) and facultatively during forward optical flow (Theobald, 2017).

Further, canopies substantially change image statistics (Dyakova et al., 2019; Schwegmann et al., 2014), creating variable levels of brightness—from heavy shade to direct sunlight. Canopy shadows in forest reduce brightness and increase spatial contrast compared to an open field (Dyakova et al., 2019; Schwegmann et al., 2014). A foraging vinegar fly thus faces a range of brightness, possibly requiring summation over short flight distances. A desert fly faces infrequent changes in brightness, lingering around a cactus, hiding in the shadow (Krebs and Bean, 1991). A necrotic cactus eventually rots out, leaving desert flies in search of another host under no canopy, requiring light adaptation that shows less sensitivity to small changes (Dyakova et al., 2019; Schwegmann et al., 2014), but still accommodating a crepuscular lifestyle.

Because many deserts are characteristically flat, bright, and barren, we offer several predictions about desert compared to vinegar flies: larger eyes, a wider FOV and horizontal streak, greater spatial acuity and lower optical and contrast sensitivity, and a less sensitive but accommodating light adaptation strategy. We predict that desert flies will have larger eyes (consistent with Keesey et al., 2019) that are specifically wider relative to body size. In addition, as eye size decreases, we predict that desert flies sacrifice contrast sensitivity more than vinegar flies to maintain horizontal FOV, spatial acuity, or both. Here, we test these predictions using a combination of microscopy, allometry, optical modeling, and—because morphology does not completely determine visual performance—psychophysics using virtual reality flight simulation. In the process, we demonstrate several visual differences that help the desert fly survive in the harsh deserts of southwest North America, while enabling vinegar flies to thrive under variably lit habitats the world over.

## Results

### General Allometry: desert flies have larger eyes

**Figure 2:**
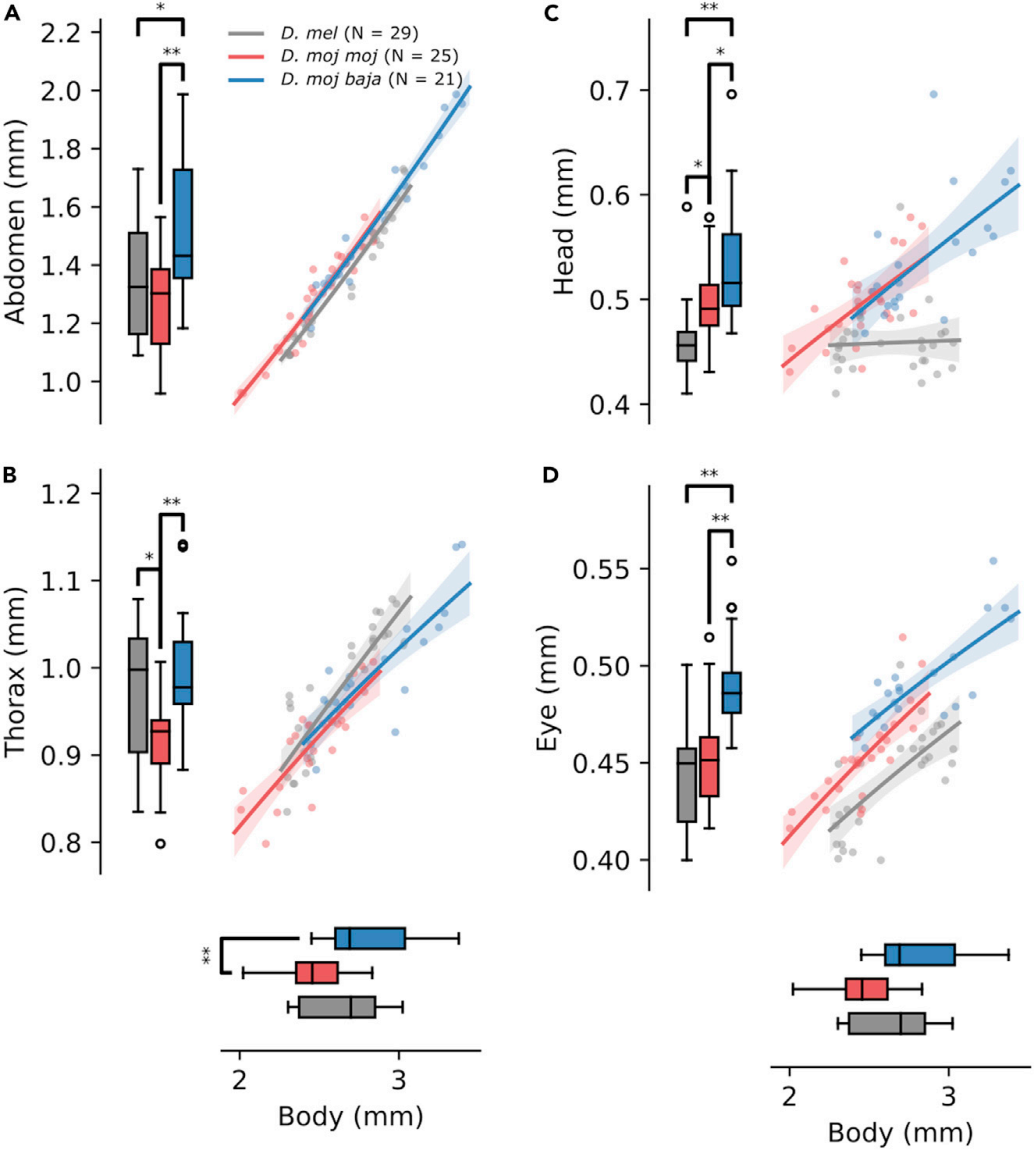
General Allometry (A–D) To measure general morphological differences between *D. mel* (gray), *D. mojmoj* (red), and *D. moj baja* (blue), we compared abdomen (A), thorax (B), head (C), and eye length (D) and their relation to body length (x axes). General differences are shown in boxplots corresponding to each axis while allometric differences are shown in the scatterplots where each dot indicates an individual fly. Each boxplot shows the sample median (black tick), IQR (upper and lower bounds of the box), range (whiskers), and outliers (fliers). Brackets point to statistically significant differences based on pairwise Student's T tests using Šidák-Holm corrected p values signified by asterisks: ∗p < .01, ∗∗p < .0001. Each scatterplot shows individual fly measurements (dots), the allometric regression mean (solid lines), and 84% confidence bands (translucent segments).

To characterize general morphological differences between the two species, we compared measurements of abdomen, thorax, head, and eye length and their allometries in relation to body length (Figure 2). Mean differences were assessed by conducting a one-way ANOVA followed by pairwise T tests using Šidák-Holm corrected p values and indicated in the boxplot comparisons of Figure 2. Relevant parameters from the allometric regressions are found in Table S1. In particular, *b* represents the growth rate of one trait, such as abdomen length, with respect to a reference trait like body length. *b* = 0 implies that *Y* is constant with respect to *X*; 0 <*b*< 1 implies hypoallometry, so that as *X* increases, *Y* increases at a decreasing rate; *b* = 1 implies isometry or linear scaling between *X* and *Y*; and *b*> 1 implies hyperallometry, so that as *X* increases, *Y* increases at an increasing rate.

We compared the vinegar fly to two desert fly subspecies: *D. mojavensis baja* from the Baja California Desert, which uses agria cactus (*Stenocereus gummosus*) as host; and *D. mojavensis mojavensis* from the Mojave Desert, which uses barrel cactus (*Ferocactus cylindraceus;* Smith et al., 2012). Despite unique host preferences, we reared all three on the same instant *Drosophila* medium. One-way ANOVAs found significant effects of genotype on abdomen (F(2,72) = 10.96, p < .001), thorax (F(2,72) = 9.81, p < .001), head (F(2,72) = 20.19, p ≪ .001), eye (F(2,72) = 25.60, p ≪ .001), and body length (F(2, 72) = 11.57, p < .001). *D. mojavensis baja* (hereafter referred to as *D. moj baja*) had a longer body, abdomen, and thorax than *D. mojavensis mojavensis* (*D. moj* *moj*), with *D. melanogaster* (*D. mel*) generally in the middle (Figures 2A and 2B). Abdomen and thorax lengths shared positive allometries with body length for all three flies, with largely overlapping confidence intervals (CIs) for the allometric constant, *b* (Figures 2A and 2B).

The relationships change for head and eye length. For the head, though *D. moj baja* was again longer than *D. moj* *moj*, *D. mel* was shorter than either desert species (Figure 2C). In addition, while allometric regressions for desert flies were similar and represented the data well, in *D. mel* neither the allometric regression nor the resulting allometric constant were statistically significant. In summary, desert fly head length was related to body length and followed the same hypoallometry, but it had no such relation in *D. mel*.

In eye length, *D. moj baja* was greater than both *D. mel* and *D. moj* *moj*, with no significant difference between *D. moj* *moj* and *D. mel* (p = .02; Figure 2D). In contrast to head length, eye length followed similar positive hypoallometries for all three flies. Nonetheless, because *D. mel* had mid-range body lengths, they had shorter eyes relative to body length (0.169 ± 0.012; not graphed) than *D. moj* *moj* (0.184 ± 0.010; p < .001) but not *D. moj baja* (0.175 ± 0.012; p = .08), with *D. moj baja* less than *D. moj* *moj* (p < .05).

Therefore, while the desert flies followed nearly equivalent positive allometries for each trait, *D. mel* did so with all but head length, which was unrelated to body length. Because head length corresponded greatly with eye width, the positive allometries suggest desert species have wider eyes that increase in width as a function of body length, while *D. mel* maintain constant eye width. Altogether, desert flies have larger eyes, and in *D. moj* *moj*, larger proportional to body length.

### Eye allometry

#### Eye shape and FOV: desert flies have a broader and particularly wider FOV

**Figure 3:**
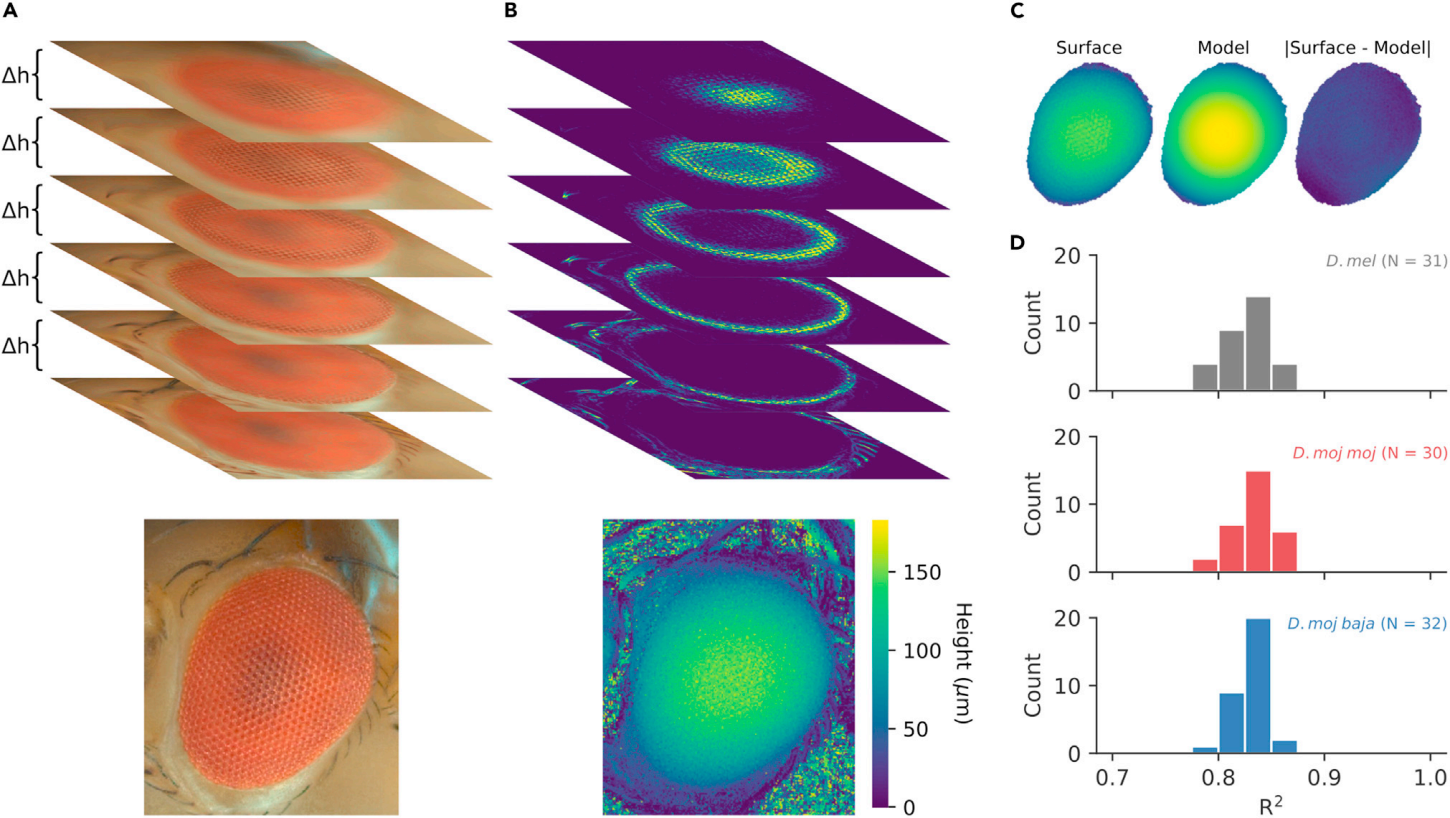
Demonstration of our method for measuring the eye radius of spherical compound eyes (A) Top: using the digital recording microscope, multiple images of the eye were taken from one angle at fixed intervals of focus depth (Δ*h*). Bottom: a focus stack was generated by taking each pixel of greatest focus (approximated by local contrast as in B.) from the stack of images. (B) By comparing measurements of local contrast for each image and finding the height that maximizes local contrast for each pixel (top), we approximated the 3D eye surface (bottom). (C) The 3D eye surface (left) was modeled as a sphere using ordinary least-squares regression (middle), resulting in small disparities between the measured and modeled values (right). The sphere provided us with an approximate radius of curvature for calculating interommatidial angles and FOV. (D) Histograms of the coefficient of determination demonstrate that the spherical model explained approximately the same proportion of the variance in eye surface values (R^2^≅ .8) for all three genotypes.

**Figure 4:**
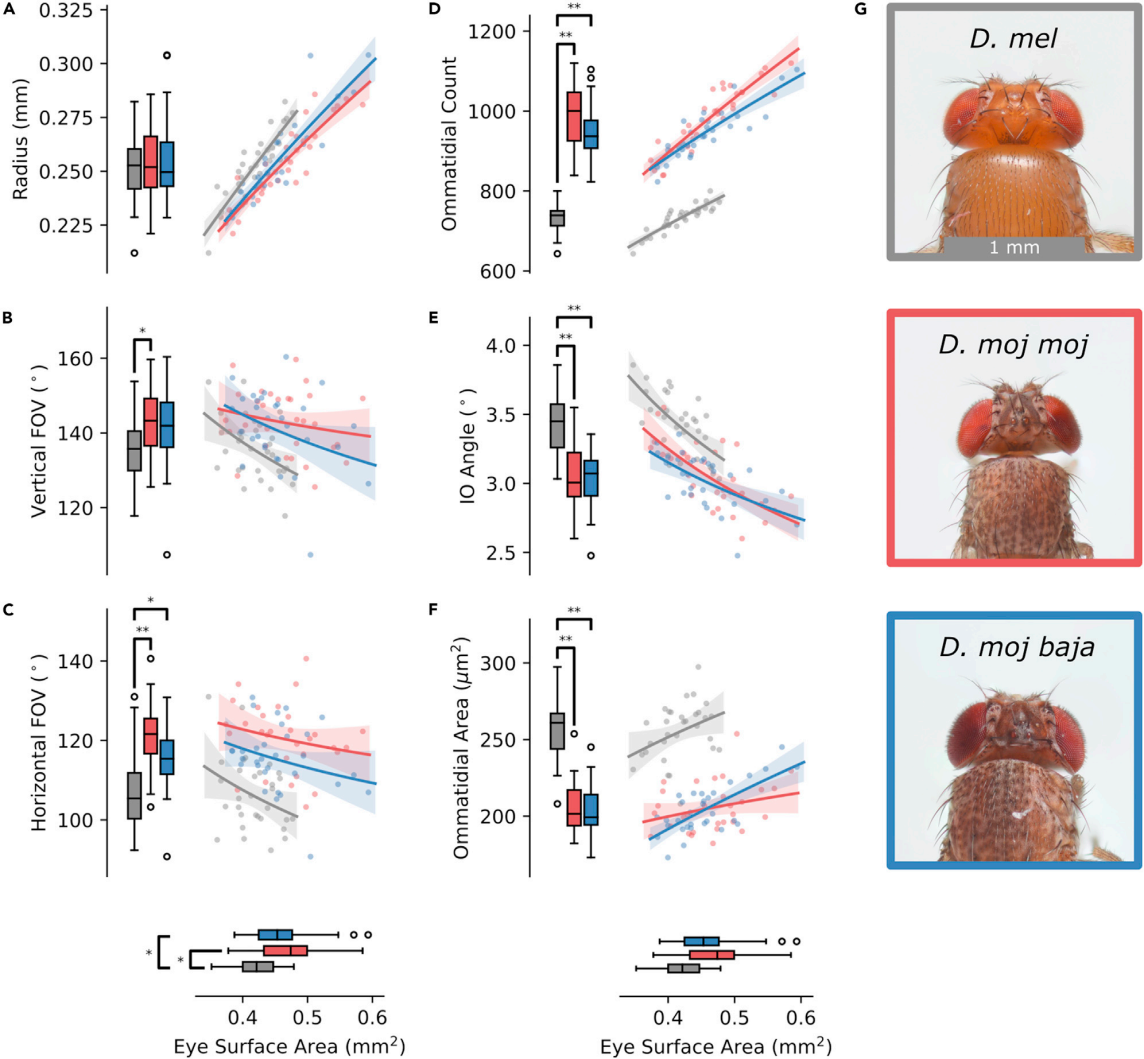
Eye Allometry (A–F) Left column: To measure visual field differences between *D. mel* (gray), *D. mojmoj* (red), and *D. moj baja* (blue), we compared eye radius (A) and vertical and horizontal FOV (B and C) and their relation to eye surface area (x axes). Middle column: for optical differences, we compared ommatidial count (D), interommatidial (IO) angle (E), and corneal lens area (F) and also their relation to eye surface area (x axes). Boxplots, significance brackets, scatterplots, and regression plots are the same as in Figure 2. (G) A dorsal view of the three genotypes shows that the posterior extreme of the eyes of desert flies (*D. mojmoj* and *D. moj baja*) recede closer to the thorax and wrap around further than vinegar flies (*D. mel*) consistent with a wider FOV. Brackets point to statistically significant differences based on pairwise Student's T tests using Šidák-Holm corrected p values signified by asterisks: ∗p < .01, ∗∗p < .0001. Each scatterplot shows individual fly measurements (dots), the allometric regression mean (solid lines), and 84% confidence bands (translucent segments). Scale bar is 1 mm. See also Figures S2 and S3.

To characterize general differences in eye structure and visual field, we compared measurements of eye area, eye radius, and vertical and horizontal components of FOV (Figures 1 and 4). Eye radius was measured by reconstructing the 3D surface of the eye by comparing images taken at known intervals of focus depth (Figure 3A). Using measurements of local contrast, a custom Python program measured for each pixel which layer—and thus height—the eye surface is in greatest focus (Figure 3B top), approximating the 3D eye surface (Figure 3B bottom). Eye radius was calculated by finding the best fitting sphere to the 3D eye surface (Figure 3C) using ordinary least squares as in Jekel (2016). The spherical model was a good fit and explains roughly the same proportion of the variance in eye surface values (R^2^≅ .8) for all three genotypes (Figure 3D). Using this center of the sphere, we convert the eye boundary to spherical coordinates, fit an ellipse to that boundary, and measure the vertical and horizontal components of FOV as the major and minor diameters of that ellipse.

One-way ANOVAs found significant effects of genotype on eye area (F(2,89) = 8.61, p ≪ .001), vertical FOV (F(2,89) = 4.90, p < .01), and horizontal FOV (F(2,89) = 20.88, p ≪ .001), but not eye radius (F(2,89) = 0.27, p = .76). *D. mel* had eyes with lower surface area than desert flies, and no significant difference within desert flies (p = .40; Figure 4, horizontal axis and boxplots; Figure S2). Conversely, eye radius showed no significant differences (Figure 4A, p > .85) and all three shared similar positive hypoallometries.

In vertical FOV (Figure 4B), *D. mel* were more restricted than desert flies, with no significant difference within desert subspecies (p = .42). Vertical FOV was independent of eye size for the desert flies but had a significant though weak negative hypoallometry in *D. mel*. In horizontal FOV (Figure 4C), *D. mel* were again more restricted than the desert flies. However, the 12° difference between *D. mel* and *D. moj* *moj* in horizontal FOV was about twice the 6° difference in vertical FOV. This is apparent in dorsal head images, revealing that the posterior extreme of desert fly eyes recede closer to the thorax and wrap around further than vinegar flies (Figure 4G). Horizontal FOV was largely independent of eye size and each allometric regression was a poor representation of the data, suggesting no significant allometries. We further analyzed vertical-to-horizontal FOV ratio (not plotted), finding a significant effect of genotype (F(2,89) = 21.20, p ≪ .001). *D. mel* had a longer eye (1.28 ± 0.06) than desert flies, *D. moj baja* (1.22 ± 0.06; p < .01) and *D. moj* *moj* (1.19 ± 0.05; p ≪ .001), with *D. moj* *moj* less than *D. moj baja* (p < .05).

In summary, the desert flies had larger eyes with the same average eye radius as *D. mel*. Eye radius increased by similar functions of eye size for all three, implying that *D. mel* had the largest radius relative to eye size. As a result, desert flies had greater, especially wider FOVs, which remained relatively constant with eye size. This confirmed the prediction of wider eyes based on general difference in head and eye length (Figures 2C and 2D), though no significant FOV allometry was found in the desert flies. Instead, each had a characteristic V/H FOV ratio independent of eye size, with the desert flies, especially *D. moj* *moj*, closer to 1.

#### Eye optics approximations: desert flies have more spatially acute but less optically sensitive eyes

We further analyzed ommatidia distribution across the visual field to approximate parameters of optical performance. Ommatidial counts and diameters were measured using the ommatidia detecting algorithm (ODA; Currea et al., 2021), which measures the number and diameter, *D*, of corneal lenses by leveraging the periodic nature of the ommatidial lattice through the use of a low-pass filter and the 2D Fourier transform. Corneal lens area, which as opposed to diameter is directly proportional to optical sensitivity, was measured as the area of a circle defined by the facet diameter, *A* = π (*D*/2)^2^. Interommatidial angle was calculated as *Δφ* = *D*/*R*, where *D* is the mean facet diameter of 300 ommatidia at the center of the eye generated by the ODA and *R* is the eye radius presented earlier. Finally, the maximum discernible wavelength was calculated as *λ*< 2*DΔφ*, assuming that lens acuity is strictly less than spatial acuity (Howard and Snyder, 1983; Snyder, 1979).

One-way ANOVAs found significant effects of genotype on ommatidial count (F(2,89) = 151.2, p ≪ .001), interommatidial angle (F(2,89) = 32.65, p ≪ .001), corneal lens area (F(2,89) = 91.94, p ≪ .001), and maximum discernible wavelength (F(2,89) = 76.85, p ≪ .001). In numbers (Figure 4D), *D. mel* had ∼255 fewer ommatidia than *D. moj baja* and ∼215 fewer than *D. moj* *moj*, with ∼40 more in *D. moj baja* than *D. moj* *moj.* The allometric regression for each was a good representation of the data, following similar hypoallometries with generally overlapping CIs for *b*. As with absolute ommatidial counts, ommatidial density (ommatidial count/eye area; not plotted) was lower in *D. mel* (1740± 75 mm⁻^2^) than in desert flies, *D. moj* *moj* (2118 ± 109 mm⁻^2^; p ≪ .001) and *D. moj baja* (2077± 121 mm⁻^2^; p ≪ .001), with no significant difference within the desert species (p = .14).

In interommatidial angle (Figure 4E), the desert flies were narrower than *D. mel*, with *D. moj* *moj* narrower than *D. moj baja*. Interommatidial angle followed a significant negative allometry with eye size in all genotypes. Conversely, in corneal lens areas (Figures 4F and S2), *D. mel* were larger than the desert flies, with no significant difference within the desert species. Lens area followed similar hypoallometries for all three with overlapping CIs for the allometric constant, though the regression was a poor fit and generated an insignificant *b* in *D. moj* *moj* and *D. mel*. Note that previous work found a significant hypoallometry in corneal lens area in *D. mel* (Currea et al., 2018). Visual inspection of micrographs (examples in the insets of Figure 1B) and scatterplots of the ommatidial centers (Figure S3) found no obvious increase in ommatidial density near the horizontal meridian, and thus no support for a horizontal streak of increased spatial acuity in any genotype.

As mentioned earlier, interommatidial angle and lens area set limitations on spatial acuity and optical sensitivity. Because desert flies have narrower interommatidial angles but smaller lenses, they have the capacity for greater spatial acuity (but at lower contrast sensitivity) than *D. mel*. Although lens area correlated positively with eye size in *D. moj baja* (Figure 4F) and previously in *D. mel* (Currea et al., 2018), interommatidial angle was negatively correlated in all flies. Consequently, as eye size increases, all flies improve spatial acuity by reducing interommatidial angle, and thus the smallest discernible details, and some improve optical sensitivity by increasing corneal lens area and thus the available light.

Finally, the maximum discernible wavelength due to diffraction (not plotted) was greater in *D. mel* (2146 ± 186 nm) than in the desert flies, *D. moj baja* (1696 ± 148nm; p ≪ .001) and *D. moj* *moj* (1722± 185nm; p ≪ .001). Further, neither the allometric regression nor the resulting allometric constants were significant for all flies (p > .07). Note that these maxima are above their range of spectral sensitivity so that neither species is operating at the diffraction limit. Instead, they imply that desert fly eyes have a lens resolution closer to their spatial resolution, indicative of eyes limited more by transduction noise than diffraction and thus evolved to operate in a brighter environment (Howard and Snyder, 1983; Snyder et al., 1977a). In particular, the effect of diffraction was independent of eye size and the eyes of the desert species were ∼80% closer to the diffraction limit than that of *D. mel*, assuming equivalent spectral sensitivities, as expected for the visual requirements of the amply lit desert.

### Psychophysics

To test the behavioral effects of spatial acuity and optical sensitivity differences described above, we measured optomotor responses to moving sinusoidal gratings in a virtual reality flight simulator equipped with a wingbeat analyzer. The difference between the left and right wingbeat amplitude (ΔWBA) indicates the fly's perceived direction of motion and is proportional to yaw torque (Götz, 1987; Tammero et al., 2004). The stimulus, a moving sinusoidal grating, is a mathematical basis for image and scene statistics, which allows us to independently manipulate contrast, temporal frequency, spatial frequency, and orientation. For each experiment, we recorded ΔWBA responses to individual gratings from a range of contrasts, spatial frequencies, and temporal frequencies, moving either left or right, sorted randomly. Then, ΔWBA was normalized to the maximum of each lighting condition, species, and experiment so that it 1) corresponds to a proportion of the maximum response of that species in that specific condition and 2) ΔWBA >0 implies a response in the direction of the stimulus and ΔWBA <0 implies countersteering. To test for significance and compare between species, normalized ΔWBA responses were averaged between .5 and 2 s, providing a mean response per fly, grating parameter, species, and experiment. These means were tested for significance and compared between species using bootstrapped confidence intervals to account for repeated measures. A grating was defined as detected if its mean ΔWBA was significantly greater than 0. Contrast sensitivity was defined as the reciprocal of the minimum detected contrast per species, spatial acuity as the maximum detected spatial frequency, and temporal acuity as the maximum detected temporal frequency preceding a >50% drop in ΔWBA. Because *D. moj baja* resisted sustained periods in the flight arena, we compared only *D. mel* with *D. moj* *moj*, which had a bigger difference in eye size and smaller but significant differences in interommatidial angle and corneal lens area.

#### Contrast sensitivity: desert flies < vinegar flies, except in low light

**Figure 5:**
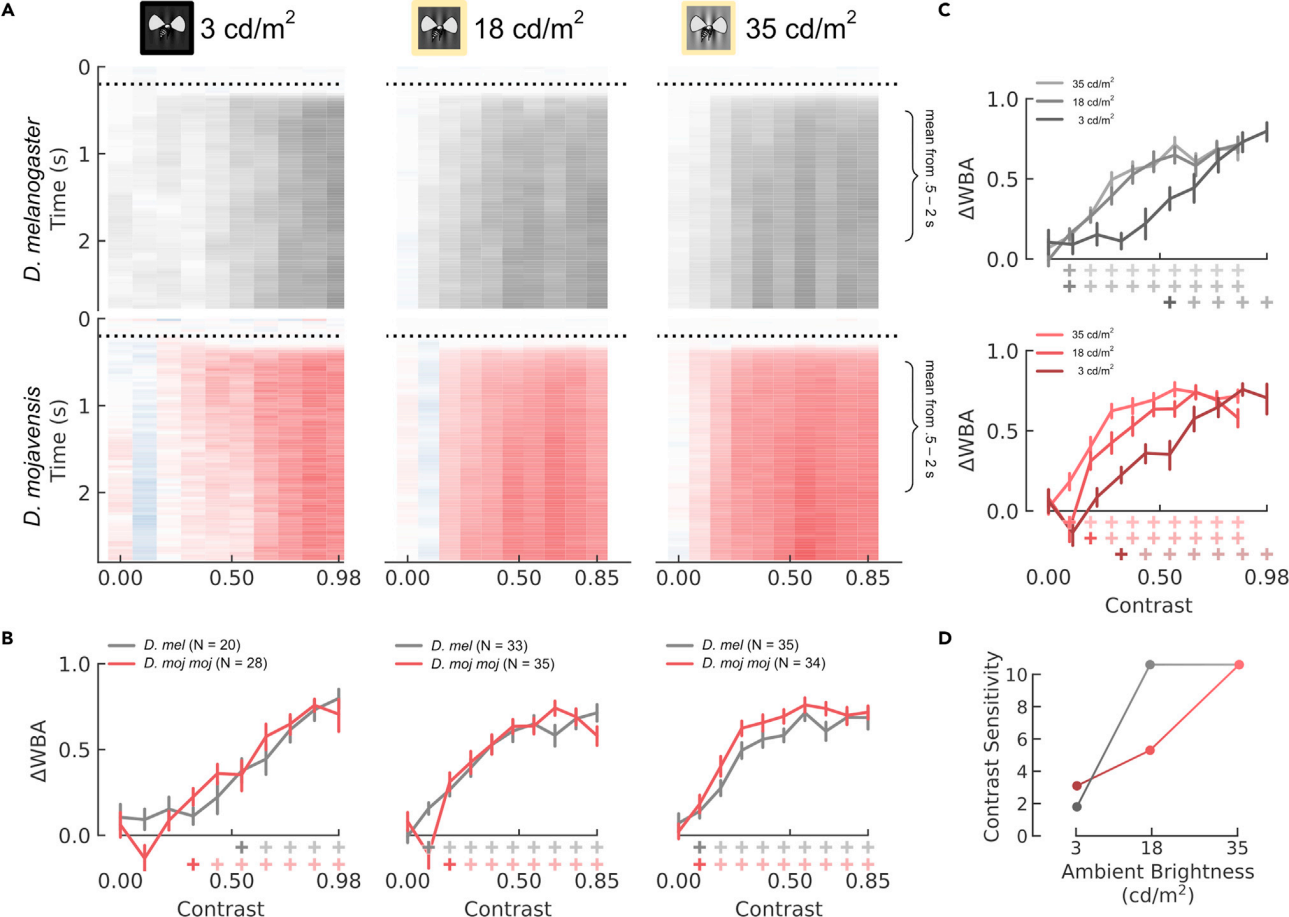
Contrast Sensitivity (A–C) Optomotor response of *D. mel* (gray) and *D. mojmoj* (red) to 10 different Michelson contrasts (x axes) in three different lighting conditions: room lights off without a neutral density (ND) filter applied (A–B, left column, 3 cd/m^2^), room lights on with an ND filter applied to the projector (column 2, 18 cd/m^2^) and without (column 3, 35 cd/m^2^). A) In each heatmap, the y axis is time from the presentation of the still grating, dotted horizontal line indicates the onset of motion, and color at each time point indicates the ΔWBA, normalized to the maximum per species (blue signifies ΔWBA <0, indicating counter steering). B–C) Mean responses are plotted in the margins below and to the right of panel A.: interspecific comparisons across lighting conditions in B. and intraspecific comparisons per lighting condition in C. Error bars indicate the standard error of the mean using bootstrapping to account for repeated measures. Plus symbols below the x axis indicate that the mean is significantly greater than 0 based on the lower bound of their bootstrapped 99% CI. Contrast sensitivity is defined as 1/C_min_, where C_min_ is the lowest Michelson contrast they respond to significantly. For each row of plus symbols, the fully saturated sign indicates C_min_. (D) Contrast sensitivity is plotted as a function of ambient brightness per species.

To measure contrast sensitivity, we presented gratings with a range of contrasts, temporal frequency of 10 Hz, and spatial frequency of .04 CPD. We define contrast sensitivity as the reciprocal of the minimum contrast responded to significantly. Because *D. moj* *moj* have significantly smaller corneal lenses, we predicted that they have lower contrast sensitivity due to reduced optical sensitivity. Nonetheless, both species detected the same minimum contrast of .09 (Figures 5A and 5B, right column, 35 cd/m^2^), implying a contrast sensitivity of 10.6 when the room lights were on and the projector was at full brightness (Figure 5D). Actually, *D. moj* *moj* had significantly greater steering responses for 4 of the 6 contrasts in the range .15–.70, suggesting a small sensitivity advantage. With a neutral density filter applied to the projector, reducing ambient brightness by ∼50% (Figures 5A and 5B, middle column, 18 cd/m^2^), *D. mel* maintained the same contrast sensitivity while *D. moj* *moj* lost detection of .09, increasing their minimum contrast to .19 and reducing their contrast sensitivity to 5.3 (Figure 5D). Finally, with lights off and the projector at full brightness (Figures 5A and 5B, left column, 3 cd/m^2^), responses changed dramatically for both species, increasing their minimum contrasts to .32 in *D. moj* *moj* and .55 in *D. mel*, dropping contrast sensitivity to 3.1 and 1.8 (Figure 5D).

These results were curious. Given their limited light absorption, how did *D. moj* *moj* achieve contrast sensitivity equal to or even greater than *D. mel* at 3 cd/m^2^? And, given the ∼50% reduction in ambient brightness, how did *D. mel* avoid losing contrast sensitivity at 18 cd/m^2^?

#### Spatial acuity: desert flies > vinegar flies, except in low light

*D. mojmoj* had significantly narrower interommatidial angles, suggesting that they have higher spatial acuity befitting their brighter environment. However, spatial acuity can be sacrificed to improve contrast sensitivity through spatial summation. To measure spatial acuity, we presented gratings with a maximum contrast (.98 when lights are off and .85 when lights are on), temporal frequency of 10 Hz, and range of spatial frequencies. We define spatial acuity as the maximum spatial frequency responded to significantly.

**Figure 6:**
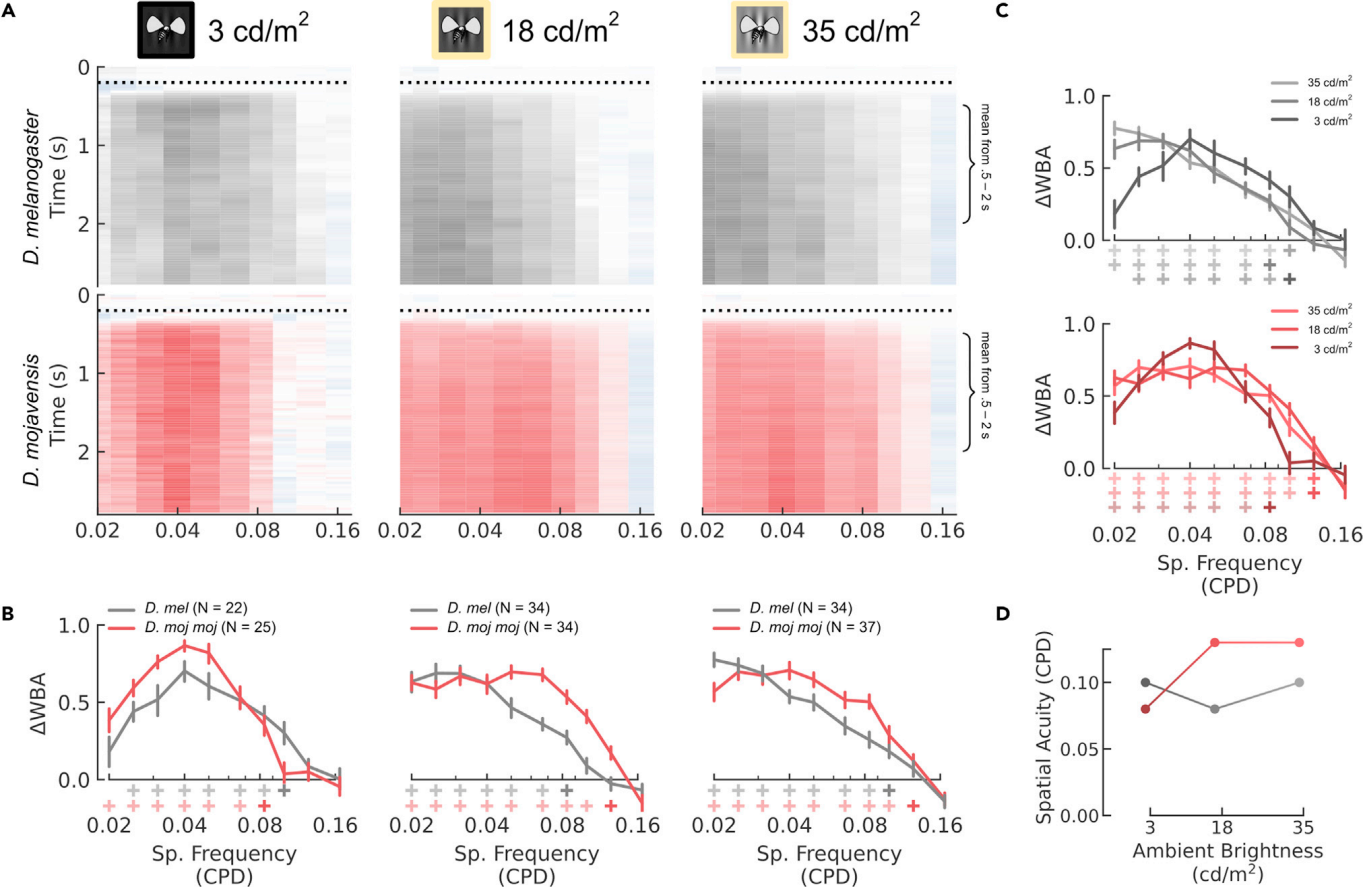
Spatial Acuity (A–C) Optomotor response of *D. melanogaster* and *D. mojavensis mojavensis* as outlined in Figure 5, but to 10 different spatial frequencies measured in cycles per degree (CPD). (B–C) Mean responses are plotted in the margins below and to the right of panel (A): interspecific comparisons across lighting conditions in (B) and intraspecific comparisons per lighting condition in (C). Error bars indicate the standard error of the mean using bootstrapping to account for repeated measures. Plus symbols below the x axis indicate that the mean is significantly greater than 0 based on the lower bound of their bootstrapped 99% CI. Spatial acuity is defined as the maximum detectable spatial frequency as indicated by the fully saturated plus symbols. (D) Spatial acuity as a function of ambient brightness per species.

Spatial acuity differed between species across all three lighting conditions, though the difference inverted under low light (Figure 6). At 35 cd/m^2^, *D. moj* *moj* had a spatial acuity of 0.13 cycles per degree (CPD) while *D. mel* had a spatial acuity of 0.10 CPD, such that *D. moj* *moj* had significantly greater steering responses than *D. mel* for spatial frequencies from 0.04 to 0.10 CPD (Figures 6A and 6B, right column). At 18 cd/m^2^, *D. moj* *moj* kept the same spatial acuity of 0.13 CPD while *D. mel* dropped to 0.08 CPD, such that *D. moj* *moj* had significantly greater steering responses than *D. mel* for spatial frequencies from 0.05 to 0.13 CPD (Figures 6A and 6B, middle column). So, *D. moj* *moj* had a greater spatial acuity at 18 and 35 cd/m^2^ (Figure 6D), which is consistent with the smaller interommatidial angles we found in *D. moj* *moj*. However, at 3 cd/m^2^, *D. moj* *moj* responded greater than *D. mel* to low spatial frequencies up to 0.05 CPD while *D. mel* responded greater than *D. moj* *moj* at 0.10 CPD (Figure 6B, left column) and spatial acuity in *D. moj* *moj* reduced to 0.08 CPD, while in *D. mel* it returned to their maximum acuity of 0.10 CPD (Figure 6D).

#### Temporal acuity: desert flies > vinegar flies, except in low light

**Figure 7:**
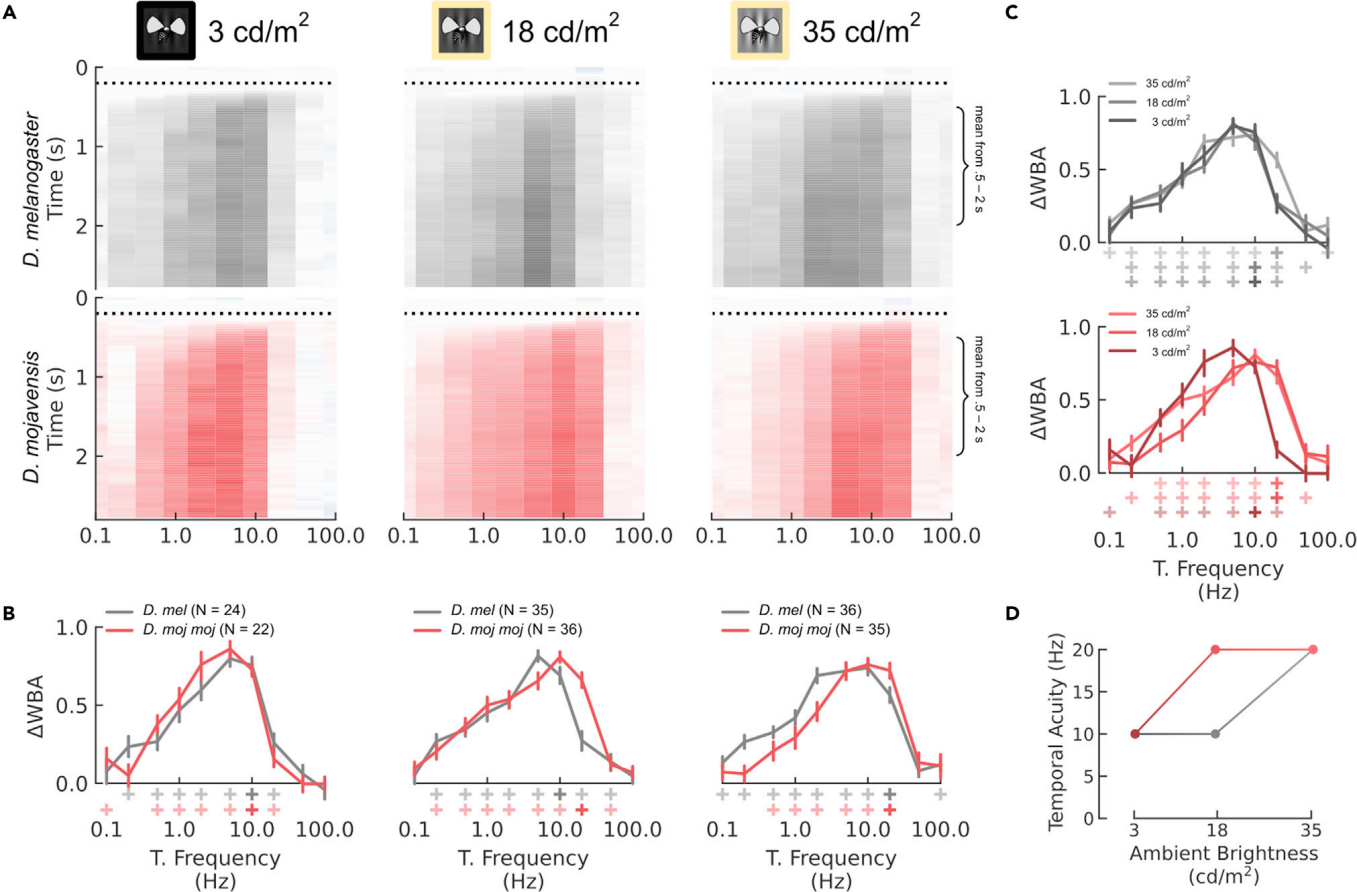
Temporal Acuity (A–C) Optomotor response of *D. melanogaster* and *D. mojavensis mojavensis* as outlined in Figure 5, but to 10 different temporal frequencies measured in cycles per second (Hz). (B–C) Mean responses are plotted in the margins below and to the right of panel A: interspecific comparisons across lighting conditions in (B) and intraspecific comparisons per lighting condition in (C). Error bars indicate the standard error of the mean using bootstrapping to account for repeated measures. Plus symbols below the x axis indicate that the mean is significantly greater than 0 based on the lower bound of their bootstrapped 99% CI. Temporal acuity is defined as the maximum temporal acuity preceding a substantial drop in response (>50%), as indicated by the fully saturated plus symbols. (D) Temporal acuity as a function of ambient brightness per species.

Because vinegar flies have larger ommatidia, they can collect the same amount of light over a shorter duration, achieving the same contrast sensitivity at a higher temporal acuity than desert flies. However, temporal acuity can be sacrificed to enhance contrast sensitivity by temporal summation in the same way as spatial acuity and summation (as in Figures 1D–1F). To measure temporal acuity, we presented gratings with a maximum contrast (.98 when lights are off and .85 when lights are on), range of temporal frequencies, and spatial frequency of .04 CPD. In addition to spatial acuity, the species also modulated temporal acuity differently under the different lighting conditions (Figure 7). Temporal acuity measurements based solely on differences of the maximum detectable temporal frequency are confounding because acuity is actually defined by the substantial reduction in contrast at higher frequencies due to insufficient sampling. In theory, this reduction in contrast is not absolute. Higher frequencies can still be detected, although sometimes oscillating in direction due to aliasing. In practice, the drop in contrast is sometimes strong enough to reduce responses to the point of statistical insignificance, such as in our spatial acuity results, though this is not always the case. For instance, the significance criterion would imply that both species have the same temporal acuity at 18 cd/m^2^ despite a clear reduction in *D. mel* responses at 20 Hz (Figures 7A and 7B, middle column). Instead, we define temporal acuity as the cutoff frequency preceding a >50% drop in ΔWBA, analogous to its half-power point.

At 35 cd/m^2^, both *D. moj* *moj* and *D. mel* had a temporal acuity of 20 Hz (Figures 7A and 7B, right column). Interestingly, *D. moj* *moj* responded greater than *D. mel* at 20 Hz and *D. mel* more than *D. moj* *moj* at frequencies less than 5 Hz, suggesting that *D. moj* *moj* have a higher optimum, which is often proportionate to temporal acuity and contrary to predictions based on their optics (Figures 7A and 7B, right column). At 18 cd/m^2^, *D. moj* *moj* showed no measurable change in responses while *D. mel* reduced temporal acuity to 10 Hz (Figures 7A and 7B, middle column). Further, *D. mel* responses were stronger at 5 Hz while *D. moj* *moj* responses were stronger at 20 Hz implying different optima. Finally, at 3 cd/m^2^, *D. mel* maintained a temporal acuity of 10 Hz while *D. moj* *moj* reduced temporal acuity to 10 Hz (Figures 7A and 7B, left column).

#### Light adaptation: sensitive vs. severe

Owing to shot noise, the signal-to-noise ratio (SNR) of light absorption is approximately the square root of the number of photons absorbed (de Vries, 1943). As a result, as light levels decrease, phototransduction SNR decreases quadratically, reducing contrast sensitivity unless spatial or temporal summation is used to increase SNR at the expense of spatial or temporal resolution. Thus, the simultaneous recovery of contrast sensitivity and loss of temporal or spatial acuity strongly implies neural temporal or spatial summation. We predict that because habitats with a canopy have more immediately variable light levels and the desert characteristically lacks a canopy, *D. mel* has a more sensitive summation strategy than *D. moj*. Nonetheless, both species are crepuscular and must fly under variable light levels likely using summation.

To measure the different summation strategies between the two species, we compared the contrast sensitivity, temporal acuity, and spatial acuity across the 3 light levels. Ambient light and projection brightness had different effects on the two species. For *D. mel*, reducing projector brightness—lowering ambient light from 35 to 18 cd/m^2^—had no measured effect on contrast sensitivity (Figure 5D, gray) but reduced spatial acuity by a relative change of 17% (Figure 6D) and temporal acuity by 50% (Figure 7D). Then, when ambient lights were off—lowering ambient light to 3 cd/m^2^— contrast sensitivity reduced by 83% (Figure 5D) but spatial acuity returned to its maximum value (Figure 6D) and temporal acuity remained the same (Figure 7D). Conversely for *D. moj* *moj*, reducing ambient light from 35 to 18 cd/m^2^ reduced contrast sensitivity by 50% (Figure 5D, red) but both spatial and temporal acuity remained constant (Figures 6D and 7D). However, reducing ambient light to 3 cd/m^2^ reduced contrast sensitivity by an additional 42% or 71% of the maximum, which is smaller than the 83% reduction in *D. mel* (Figure 5D), spatial acuity by 33% (Figure 6D), and temporal acuity by 50% (Figure 7D). Thus, light adaptation in *D. mel* was more sensitive to small brightness changes while in *D. moj* *moj*, spatial and temporal summation were used more severely but only after a large reduction in ambient light.

## Discussion

These observations resolve many of our earlier predictions. First, the terrain theory posits that animals from open-country terrains evolve a wide FOV and horizontal streak of increased spatial acuity (Hughes, 1977). While we found no evidence for the horizontal streak (Figures 1B, zoomed insets and S3), desert flies have a larger eye with a particularly wider FOV, corroborating that desert flies allocate more resources to vision than vinegar flies (Keesey et al., 2019) and satisfy the FOV prediction of terrain theory. Interestingly, neither horizontal nor vertical FOV followed an allometric relation to body size within species, except a slight negative hypoallometry in vinegar flies, suggesting that FOV faces strong but different selective pressures for both flies (Wehner et al., 2014). As mentioned earlier, evolution of the visual streak depends on a visible horizon, and as both flies are crepuscular, the effect may be weak.

Instead, the streak could manifest neurally by differential spatial summation across the eye. In fact, vertebrate visual streaks result from spatial pooling of retinal ganglion cells (Hughes, 1977). Similar regional spatial summation would be externally invisible and could be facultative (Theobald, 2017), making regional acuity sacrifices only when needed, such as during foraging but not courtship. Alternatively, like Australian desert ants (*Melophorus bagoti*; Schwarz et al., 2011), desert flies might be an exception to terrain theory, based on unknown environmental factors or lifestyle. Future work could measure spatial acuity differences across the visual field to test for neural implementations of horizontal streaks.

Next, because deserts are characteristically bright, we predicted desert flies' eyes are less sensitive, more acute, and closer to the diffraction limit (Snyder et al., 1977a, 1977b; Snyder, 1979). These were confirmed with eye morphology, revealing smaller ommatidia separated by narrower interommatidial angles in desert flies than vinegar flies, and implying a lower maximum discernible wavelength. We also predicted that small desert fly conspecifics may sacrifice optical sensitivity (*D* in Figure 1C) more than vinegar flies in order to minimize losses in spatial acuity (1/*Δφ*) and FOV. However, all species followed similar hypoallometries for corneal lens area implying that their eye development faces similar selective pressures. Some of these observations were consistent with optomotor behavior in the flight arena, where *D. moj* *moj* demonstrated higher spatial and temporal acuities but lower contrast sensitivity under moderate light (18–35 cd/m^2^). Because both species are active at similar times (Hardeland and Stange, 1973; Keesey et al., 2019), this may be due to the lack of shade from vegetation or terrain relief in many deserts.

Fewer desert shadows generate a less immediate brightness range than habitats of the cosmopolitan vinegar fly, but both flies are crepuscular, requiring sensitivity to the large, gradually changing brightness range of sunrise and sunset. We predicted desert fly vision is less susceptible to small changes in ambient brightness but still responsive to large changes and tested this by performing flight arena experiments under 3 different light levels (3, 18, and 35 cd/m^2^). We found desert flies make greater spatial and temporal acuity sacrifices than vinegar flies to maintain contrast sensitivity only after a large reduction in ambient light (from 18 to 3 cd/m^2^). Spatial summation found in desert flies at the lowest brightness (3 cd/m^2^) may be implemented by laterally extended dendrites of the lamina monopolar cells or electrical coupling of photoreceptors in individual ommatidia (Dubs et al., 1981; Greiner et al., 2005; Stöckl et al., 2020; Warrant et al., 2004). This strategy of less sensitive but more severe summation affords desert flies high spatiotemporal acuity under moderate light, like when foraging for a cactus on the horizon, but permits adequate contrast sensitivity when light is sparse, like during courtship, which occurs in the early morning shadow of a host cactus (Krebs and Bean, 1991). Future work could measure natural light levels during behaviors like foraging, as little is known about how desert flies forage for new cactus hosts.

In addition, while our range of brightnesses, from 3 to 35 cd/m^2^, does include many of the crepuscular brightness values they fly under, they do not represent the full range of brightnesses either species is exposed to. More interesting changes might occur under brighter light, like improving contrast sensitivity or spatial or temporal acuity through the release of additional neural summation, but this was ultimately limited by our projector. Alternatively, as light levels fall below 3 cd/m^2^, desert flies may 1) spatially or temporally summate further to recover contrast sensitivity, like vinegar flies do generally (Palavalli-Nettimi and Theobald, 2020) and in response to reduced growth (Currea et al., 2018), 2) sustain losses in contrast sensitivity, or 3) slow down, walk, or stop in order to keep spatial and temporal frequencies within the detectable range. In our lab, desert flies were less likely to fly in general and, in the field, desert fly courtship was not observed until shortly after sunrise and was unobserved around sunset (Krebs and Bean, 1991), supporting possibility #3 that desert flies move more slowly or less often under dimmer light. Future work should characterize the summation strategies of desert versus vinegar flies to the full range of light levels they might fly under and apply electrophysiological techniques to identify the mechanisms of spatial and temporal summation in the desert fly.

In addition to comparing vinegar and desert flies, we measured morphological differences between desert subspecies. When reared on standard media, both *D. moj* *moj* and *D. moj baja* follow nearly equivalent allometries for body and thorax length, though *D. moj baja* are generally larger (Figure 2). Because *D. moj baja* are smaller than the Sonoran Desert subspecies (*D. mojavensis sonorensis*; Etges and Heed, 1987), *D. moj* *moj* is smaller than at least 2 of the 3 known *D. mojavensis* subspecies. Still, *D. moj* *moj* and *D. moj baja* eyes are similar in size and allometries for surface area, radius, vertical and horizontal FOV, ommatidial count, interommatidial angle, and corneal lens area (Figure 4). In summary, despite general size differences, desert fly eyes likely follow similar developmental processes regarding cellular proliferation and growth during the formation of the eye-antennal imaginal discs (Currea et al., 2018) with *D. moj* *moj* making a greater relative investment in vision than *D. moj baja*.

For desert flies, the necrosis chemistry of different cactus species differentially affects gene expression (Matzkin et al., 2006), development time, viability, and overall size (Etges and Heed, 1987). So, because *D. moj baja* target the Pitaya agria cactus (*Stenocereus gummosus*) and *D. moj* *moj* target California barrel cactus (*Ferocactus cylindraceus;* Smith et al., 2012), but we reared both on standard *Drosophila* media, the ecological validity of these comparisons is speculative. Cactus stem diameter affects desiccation and correlates with total rot duration and subsequently thorax size across many cactophilic species including *D. moj,* and between the subspecies of *D. moj baja* and *D. mojavensis sonorensis* (Etges and Heed, 1987). *D. mojavensis sonorensis* are larger than both *D. moj* *moj* and *D. moj baja* and use organ pipe cactus (*Stenocereus thurberi*) which have a mid-range stem diameter of 5–20 cm (Anderson, 2001). So, if our results reflect natural size distributions, then *D. mojavensis* are an exception to this positive correlation between stem diameter and body size: California barrel cactus is ≤50 cm in stem diameter but hosts the smaller *D. moj* *moj* while Pitaya agria cactus is 3–6 cm but hosts the larger *D. moj baja* (Anderson, 2001; Smith et al., 2012). Future work should investigate how specific resources like medium composition and temperature, which influence allometry in holometabolous insects (Callier and Nijhout, 2013; Shingleton et al., 2007; Stern and Emlen, 1999) and cactophilic flies in particular (Etges and Heed, 1987; Etges and Klassen, 1989), affect eye development and subsequently visual performance across desert flies.

### Conclusion

Are the desert fly visual traits we found ancestral and lost by the unique evolution of the vinegar fly, or a unique adaptation to cactophily, as suggested by the terrain hypothesis? Because the vinegar fly belongs to the *Drosophila* (*Sophophora*) subgenus and the desert fly to the more derived *Drosophila* (*Siphlodora*) subgenus (O'Grady and DeSalle, 2018; Yassin, 2013), parsimony suggests that differences between the desert and vinegar fly represent derived characters of members of the *Sipholodora* subgenus. In fact, the *D. repleta* species group containing desert flies represents a natural group demonstrating genetic innovations among *Drosophila* that are likely adaptations to the cactophillic lifestyle, aiding in the identification of the appropriate host plant and tolerance of the toxic chemicals produced by cactus necrosis (Markow, 2019; Wasserman, 1960). Phylogenetic analyses therefore support the conclusion that the visual differences we have demonstrated are due to the unique natural history of desert flies.

However, further species comparisons are needed to understand the particular evolution of these traits. The *D. repleta* species group offers a unique system for probing these questions because *D. mojavensis* and the other North American *repleta* species diverged from South American relatives about 12 MY ago (Markow, 2019; Smith et al., 2012). The natural replication of cactophilic flies in the Americas—such as *D. buzzatii* which uses prickly pear cactus (*Opuntia*) as host—offers a quasi-experiment in the evolution of these traits. In particular, the *D. mojavensis* and *D. buzzatii* genomes have been sequenced (Guillén et al., 2019; Markow, 2019), allowing for additional genetic analyses.

Despite these unknowns, desert flies have larger eyes, a wider FOV, and more, smaller ommatidia than vinegar flies, fitting several predictions based on characteristics of their desert environment. Owing to the critical role of neural summation for light adaptation, inferences based on eye morphology depend on features of the visual environment, and our results highlight the importance of studying behavior across different ambient light levels. In doing so, we found visual differences that help desert flies survive in the flat, bright, and barren desert while accommodating their crepuscular and biphasic adult lifestyle. Meanwhile, vinegar flies' more sensitive but less severe light adaptation allows them to see under more immediately variable light levels around the world.

### Limitations of the study

Our study faced several limitations. 1) While our range of brightnesses, from 3 to 35 cd/m^2^, does include many of the crepuscular brightness values they fly under, they do not represent the full range of brightnesses either species is exposed to. More interesting changes might occur under brighter light but this was ultimately limited by our projector. 2) Our comparison only included one desert species, with two subspecies, and the vinegar fly. Further species comparisons would clarify the particular evolution of the visual differences we measured. 3) Many of these comparisons relied on morphology, which as we showed is only a partial determinant of visual performance. To test for differences in spatiotemporal acuity due to neural processing across the visual field like the visual streak, behavioral or electrophysiological methods are needed. 4) Likewise, our spherical model of the eye is only an approximation. More detailed imaging methods, such as micro-CT, would allow for more precise morphological and allometric comparisons.

## STAR★Methods

### Key resources table

**Table:** 

REAGENT or RESOURCE	SOURCE	IDENTIFIER
**Deposited data**		

General Allometry Dataset and Code	https://figshare.com/	https://doi.org/10.6084/m9.figshare.15127776.v1
Eye Allometry Dataset and Code	https://figshare.com/	https://doi.org/10.6084/m9.figshare.15127797.v3
Psychophysics Dataset and Code	https://figshare.com/	https://doi.org/10.6084/m9.figshare.15130422.v1

**Software and algorithms**		

Compound Eye Tools API	John Paul Currea on github.com	https://github.com/jpcurrea/eye_tools/

### Resource availability

#### Lead contact

Further information and requests for resources should be directed to and will be fulfilled by the Lead Contact, Jamie Theobald (theobald@fiu.edu).

#### Materials availability

This study did not generate new unique reagents.

### Experimental model and subject details

*Drosophila melanogaster* (Meigen) were drawn from a colony reared in laboratory conditions for several years. *D. mojavensis mojavensis* and *D. mojavensis baja* were sent from the Garrity Lab at Brandeis University and subsequently raised under the same protocols. For all experiments, wild-type flies from the three genotypes were reared at 21.5°C, under a 12 hour light:12 hour dark cycle, and on Carolina Formula 4-24 Instant *Drosophila* Medium, Plain. We randomly selected individuals for each genotype, 2-5 days post eclosion, with males and females in roughly equal proportions. 2-5 post eclosion flies give robust behavioral performance, and using both male (smaller) and female (larger) flies gives the greatest size range for allometry. Different samples were used for the psychophysics and morphological experiments, allowing independent tests of our hypotheses about contrast sensitivity and spatial acuity. Because the psychophysical experiments were time demanding, we did not also measure their morphology. As a result, the size distribution should be approximately the same across both experiments given that all subjects were reared under the same conditions and selected via the same process.

### Method details

#### General allometry

Subjects were drawn from each genotype 3–6 days post eclosion and were euthanized by placing them in a freezer for 48 hours. Next, they were glued to a rigid tungsten rod, 0.02 mm in diameter, on the dorsal prothorax for easy manipulation and were photographed laterally using a digital recording microscope (Zeiss Axio Scope.A1). Trait lengths were measured manually on the microscope images using a custom Python program (Figure 1A). Eye length was measured as the longest line through the eye, $\bar{ab}$, which was approximately vertical and perpendicular to the head length. To account for idiosyncratic bending of the abdomen or head, body length was measured as the sum of the lengths of the three body segments. The head segment was measured from the dorso-anterior tip of the antenna flagellum, *c*, to the anterior tip of the head occiput, near the center of the neck and often coinciding with anterior tip of the eye, *d*. The thorax segment was measured from *d* to the base of the haltere, *e.* The abdomen segment was measured from *e* to the posterior tip of the abdomen, *d*. The more conventional thorax length was taken from the anterior margin at the neck (*g*) to the posterior tip of the scutellum (*h*; Atkinson and Sibly, 1997; Morin and Moreteau, 1996). We analyzed how abdomen ($\bar{ef}$), thorax ($\bar{gh}$), head ($\bar{cd}$), and eye lengths ($\bar{ab}$*)* scale with respect to body length ($\bar{cdef}$; Figure 1A). The values needed to compare these allometries are available in Table S1.

#### Eye allometry

For eye measurements, live adults were drawn from the 3 genotypes around 3-6 days post eclosion and were cold-anesthetized and glued to a rigid tungsten rod, 0.02 mm in diameter, on the dorsal prothorax. Flies from *D. melanogaster* and *D. mojavensis mojavensis* were then placed in the flight arena for vision experiments described below. Flies from *D. mojavensis baja* had difficulty maintaining flight and thus were excluded from our behavioral experiments.

Next, glue was applied to the neck, adhering the head to the thorax to avoid head motion during imaging. Using the digital recording microscope, multiple images (∼20) were taken per fly from one angle, at fixed intervals of focus depth (Figure 3A). This allowed us to approximate the eye surface as a sphere for comparisons of optical parameters.

#### Psychophysics

##### Flight arena

The flight arena consisted of a back-projection lined perspex cube with four first-surface mirrors angled to project a stimulus onto five of its six sides (Figure 1G). For more details see (Cabrera and Theobald, 2013). The current study used only the front ± 45° azimuth and ± 45° or ± 67.5° elevation, mostly occupying the front 90 X 90°, 229 X 229 pixel panel. At the center of the arena, each tethered fly tries to minimize the perception of motion, but is left immobilized while still beating its wings (Götz, 1987; Tammero et al., 2004). An infrared LED, which is invisible to and placed above the fly, casts a shadow of each wing onto a corresponding photodiode below. The photodiodes provide a time series of the fly's left and right wing beat amplitude (ΔWBA) at 1 kHz. The difference between the left and right ΔWBA indicates the fly's perceived direction of motion and is proportional to yaw torque (Figure 1H: data sample; Götz, 1987; Tammero et al., 2004). For all experiments using the flight arena, ΔWBA was normalized to the maximum of each lighting condition, species, and experiment so that it corresponds to a proportion of the maximum response in that specific condition.

##### Lighting conditions

Flight arena experiments were conducted under three different lighting conditions. Mean luminance and maximum contrast were measured in each condition using a Gossen Starlite 2 light meter. Luminance measurements were taken by placing the hemisphere sensor at the position of the fly, centered in the arena and pointed towards the front panel. Maximal contrasts were determined previously by luminance measures of the brightest and darkest points of the projection in the arena (Caballero et al., 2015; Cabrera and Theobald, 2013). Initially, flies were tested in a dark room, with the lights off. This resulted in a maximum Michelson contrast of 98% and an average brightness of 3 cd/m^2^ inside the arena. Later, a different sample of flies was tested in a room with the lights on and with or without a neutral-density filter applied to the projector. The neutral-density filter reduced the brightness of the projection by roughly an order of magnitude (∼90%). Having the room lights on resulted in a maximum Michelson contrast of 85% and an average brightness of 18 cd/m^2^ inside the arena with the filter applied to the projector and 35 cd/m^2^ without the filter. To compare psychophysical results across lighting conditions, we define the relative change from measurement A to B as $\frac{A-B}{A}$.

##### Gratings

In the arena, flies were presented a series of experiments to measure the visual consequences of their eye structure. Experiments consisted of open-loop sequences of moving sinusoidal gratings interspersed by 3 s bouts of closed-loop fixation of a vertical striped bar. During fixation, the fly controlled the position of the striped bar, improving their responsiveness to experimental stimuli (Heisenberg and Wolf, 1979; Reichardt and Wenking, 1969). During test sequences, grayscale sinusoidal moving gratings from a list of spatial frequencies, temporal frequencies, and contrasts, moving either to the left or right, were presented in a randomized order. The grating remained still for the first .2 s to use for baseline comparisons (indicated by dotted lines in Figures 5, 6, and 7). Each grating was presented for about 3 s, followed by the fixation task, until each fly was exposed to the whole list. For experiments with lights on, whenever possible, flies were randomly assigned to view all experiments in one projector condition followed by the opposite.

For experiments with the lights off, gratings were filtered through a gaussian window, covered a 60° solid angle, and centered at either -45, 0, or 45° elevation. Initially, the experiments with lights off were designed to measure both general visual differences and specific differences below, at, or above the horizon. However, the results were confounded by likely differences in head position and FOV and instead were averaged across the 3 positions to measure general differences. For experiments with lights on, gratings passed through a gaussian window, covered a 90° solid angle, and were centered at the horizon.

### Quantification and statistical analysis

#### Eye morphology

Using the digital recording microscope, multiple images (∼20) were taken per fly from one angle, at fixed intervals of focus depth (Figure 3A). Using measurements of local contrast, we can measure for each pixel which layer—and thus height—the eye surface is in greatest focus, allowing us to reconstruct the 3D eye surface (Figure 3B). Eye radius was calculated by finding the best fitting sphere to the 3D eye surface (Figure 3D) using ordinary least squares as in Jekel (2016). To apply ordinary least squares, start with the following equation for the radius of a sphere with points at (***x***, ***y***, ***z***), a center at (*x*_*0*_, *y*_*0*_, *z*_*0*_), and radius *r*:

(Equation 1) $${\left(x-x_{0}\right)}^{2}+{\left(y-y_{0}\right)}^{2}+{\left(z-z_{0}\right)}^{2}=r^{2}$$

Bold variables indicate 1-D vectors. Equation 1 can be expanded and rearranged into the following:

(Equation 2) $$x^{2}+y^{2}+z^{2}=2xx_{0}+2yy_{0}+2zz_{0}+r^{2}-{x_{0}}^{2}-{y_{0}}^{2}-{z_{0}}^{2}$$

Then, define the following matrices in order to express Equation 2 in matrix notation:

(Equation 3) $$f=\left[x^{2}+y^{2}+z^{2}\right]$$

(Equation 4) A=[2x,2y,2z,1]

(Equation 5) $$c={\left[x_{0},y_{0},z_{0},\;r^{2}-{x_{0}}^{2}-{y_{0}}^{2}-{z_{0}}^{2}\right]}^{\text{T}}$$

Substituting Equations 3, 4, and 5 into Equation 2 gives ***f*** = *A****c***. Finally, apply ordinary least squares to find the vector ***c*** that minimizes the squared deviation between ***f*** and *A****c***, approximating the center of the sphere, (*x*_0_, *y*_0_, *z*_0_). With that center, we calculate the mean radius as:

(Equation 6) $$r=\text{mean}\left(\sqrt{\left[{\left(x-x_{0}\right)}^{2}+{\left(y-y_{0}\right)}^{2}+{\left(z-z_{0}\right)}^{2}\right]}\right)$$

This method was verified on 3D digital image correlation reconstructions of the surface of bearing balls, finding an error of .0023–.0067 (Jekel, 2016). To validate it using our focus stack pipeline, we applied the program to glass homogenizing beads with a radius of 50 ± 5 (mean ± s.e.), 250 ± 25, and 500 ± 50 μm from BioSpec Products, Inc. (Figure S1). We tested 5 beads for each radius and used ordinary least squares to model the product radius as our automated measurements times a scalar. The regression model was a good fit (R^2^ = .98, p ≪ .001) and the product radius is approximately 109 ± 4% times the measured radius (t = 28, p ≪ .001), suggesting that this method offers a reliable approximation of the radius of curvature.

Using the center of the sphere, the coordinates were spherically transformed, allowing us to generate a flattened image of the eye correcting its curvature (Figure 3D). FOV was calculated as the area of a best fitting ellipse to the outline of the eye in spherical coordinates and its vertical and horizontal components as the major and minor diameters. Note that distance and area in spherical coordinates correspond to angle and solid angle in cartesian coordinates.

To measure optically relevant parameters, an open source Python program (Currea et al., 2021) generated a single image composite of the stack of photos (Figure 3A, bottom). Ommatidial counts and diameters were measured using the ommatidia detecting algorithm (Currea et al., 2021), which measures the number and diameter, *D*, of ommatidia by using the fast fourier transform to generate the reciprocal image of the focus stack image (Figure S2). In the reciprocal image, each pixel represents a sinusoidal grating in the original image with a particular contrast (the pixel’s value), spatial frequency (its reciprocal distance from the center) and orientation (its polar angle). The reciprocal image of a hexagonal lattice is itself a hexagonal lattice, which can be detected by applying a 2D autocorrelation (Figure S2, grayscale images). By finding the fundamental frequencies of the reciprocal image (maxima closest to the center), the ODA applies a low-pass filter to the original image to more easily detect the center of ommatidia (Figure S3). The ODA has been validated on the eyes of *D. melanogaster*, generating facet diameters and counts equal to 105 ± 3% and 104 ± 4% and with correlations of .93 and .82 of those measured manually (Currea et al., 2021). The spherical model was also a good fit and explained roughly the same proportion of the variance in eye surface values (R^2^≅ .8) for all three genotypes (plotted in Figure 3D).

Interommatidial angle was calculated as *Δφ* = *D*/*R*, where *D* is the mean facet diameter of 300 ommatidia at the center of the eye generated by the ODA and *R* the eye radius generated by the focus stack method mentioned above. Corneal lens area, which as opposed to diameter is directly proportional to optical sensitivity, was measured as the area of a circle defined by the facet diameter, *A* = π (*D*/2)^2^. Finally, the maximum discernible wavelength was calculated as λ< 2*DΔφ*, assuming that lens acuity is strictly less than spatial acuity (Howard and Snyder, 1983; Snyder, 1979). The values needed to compare these allometries are available in Table S2.

#### Allometry

The scaling relation between physical traits, called allometry, helps quantify environmental reaction norms. They are usually modeled as a power law, *Y* = *aX*^*b*^, where *Y* and *X* are traits and *a* and *b* are constants (Shingleton et al., 2007; Voje et al., 2014). The allometric constant, *b*, represents the growth rate of *Y* with respect to *X*. Given *a*> 0, *b* = 0 implies that *Y* is constant with respect to *X*; 0 <*b*< 1 implies hypoallometry, so that as *X* increases, *Y* increases at a decreasing rate; *b* = 1 implies isometry or linear scaling between *X* and *Y*; and *b*> 1 implies hyperallometry, so that as *X* increases, *Y* increases at an increasing rate. *a*< 0 implies the same relations except with an inverse allometry.

To apply this model, we log transformed both traits *X* and *Y* and then used ordinary least squares regression to model log(*Y*) as a function of log(*X*), *log*(*Y*)=*log*(*a*)+*b*⋅*log*(*X*). 84% confidence bands, used for comparing parameter means between species with ɑ = .05 (Goldstein and Healy, 1995), were approximated by applying the regression model in log space and then exponentiating *e* to the power of the resulting estimates (shaded areas in Figures 3 and 4, for example).

#### Morphological comparisons

Morphological measurements were first compared by a one-way analysis of variance (ANOVA) and post-hoc pairwise T tests using Šidák-Holm corrected p-values. The significance of these comparisons is indicated in the box plot comparisons of Figures 3 and 4. These measurements were then compared in terms of how they scale in relation to body or eye size.

#### Behavior

To measure if a particular grating condition was detected, we conducted a similar analysis as in Currea et al. (2018). Each grating was displayed moving once to the left and once to the right. ΔWBA responses were normalized and combined so that ΔWBA > 0 indicates a correct response and ΔWBA < 0 indicates movement opposing the stimulus motion. Responses were also normalized to the maximum response of each particular genotype and psychophysics experiment (contrast, temporal frequency, or spatial frequency). Because fly responses were usually delayed by about .3 seconds, ΔWBA values were averaged from .5 to 2 s after presentation or .3 to 1.8 s after the onset of motion. Standard error and 99% CI of the mean for each condition within an experiment were found using bootstrapping to account for repeated measures. Error bars in the marginal plots of Figures 5, 6, and 7 represent this bootstrapped standard error and allow for mean comparisons between species. Plus symbols below the x-axis of each marginal plot in Figures 5, 6, and 7 indicate that the mean is significantly greater than 0 based on the lower bound of their bootstrapped 99% CI. Therefore, a grating is considered detected by that species if the lower bound of the bootstrapped 99% CI is greater than zero and is indicated by a plus symbol below the x-axis.

The moving sinusoidal grating is ideal for measuring optical performance. It allows the independent manipulation of contrast, spatial frequency, and temporal frequency while maintaining the same mean luminance across all conditions. Contrast is defined as the Michelson contrast, which is the difference between the brightest and darkest intensities of the grating divided by their sum. Contrast sensitivity is measured as the inverse of the lowest detected contrast. Spatial frequency defines the frequency of luminance change over distance in cycles / ° (CPD). Spatial acuity is measured as the highest detected spatial frequency (Land, 1997; Land and Nilsson, 2012). Temporal frequency defines the frequency of luminance change over time in cycles per second or Hz. Though temporal acuity is often measured as the highest detected temporal frequency, an accurate and complete description of acuity includes relative ability to respond at different frequencies. Here temporal acuity is defined as the temporal frequency that precedes a significant and substantial (>50%) drop in ΔWBA.

## Data Availability

• All data and code for generating the figures and statistics used in this study are publicly available on figshare.com, with DOIs listed in the key resources table. • The Compound Eye Tools API used for detecting ommatidia in an image is publicly available on github.com (https://github.com/jpcurrea/eye_tools/) and detailed in a preprint (Currea et al., 2021). • Any additional information required to reanalyze the data reported in this paper is available from the lead contact upon request.
