# Supplementary material for: Acuity and summation strategies differ in vinegar and desert fruit flies
Source: iScience. 2021 Dec 16;25(1):103637. doi: 10.1016/j.isci.2021.103637 (PMC8741510; doi:10.1016/j.isci.2021.103637)
Supplement: Document S1. Figures S1–S3 and Tables S1 and S2 [file mmc1.pdf]

iScience, Volume *25*

Supplemental information

Acuity and summation strategies differ in vinegar and desert fruit flies

John P. Currea, Rachel Frazer, Sara M. Wasserman, and Jamie Theobald

Supplemental Information

Trait	Species	R^2	*b*	84% C.I. for *b*
Abdomen	*D. mel*	.96**	1.47**	(1.39, 1.56)
	D. moj moj	.91**	1.44**	(1.30, 1.58)
	D. moj baja	.96**	1.42**	(1.32, 1.52)
Thorax	*D. mel*	.76**	0.67**	(0.57, 0.78)
	D. moj moj	.69**	0.55**	(0.44, 0.66)
	D. moj baja	.70**	0.52**	(0.40, 0.63)
Head	*D. mel*	<.01	0.03	(-0.15, 0.22)
	D. moj moj	.43*	0.58*	(0.24, 0.35)
	D. moj baja	.44*	0.66*	(0.21, 0.35)
Eye	*D. mel*	.48**	0.42**	(0.30, 0.54)
	D. moj moj	.64**	0.46**	(0.36, 0.57)
	D. moj baja	.64**	0.37**	(0.28, 0.46)

Symbol	Meaning
*	p < .01
**	p < .0001

Table S1: Parameters from allometric regressions of body measurements, related to STAR methods. Asterisks indicate the level of statistical significance of the corresponding R^2 or *b* based on the small table at the bottom. 84% confidence intervals (C.I.) of *b* allow for comparisons of the allometric constant between species by considering their overlap. Finding that the C.I.'s do not overlap is statistically equivalent to a Student's T-test with $\alpha < .05$ (Goldstein and Healy 1995).

Outcome Trait	Species	R^2	*b*	84% C.I. of *b*
Radius	*D. mel*	0.78**	0.679**	(0.58, 0.78)
	D. moj moj	0.82**	0.561**	(0.49, 0.63)
	D. moj baja	0.79**	0.607**	(0.52, 0.69)
Vertical FOV	*D. mel*	0.22*	-0.35*	(-0.53, -0.17)
	D. moj moj	0.04	-0.106	(-0.25, 0.04)
	D. moj baja	0.11	-0.244	(-0.43, -0.06)
Horizontal FOV	*D. mel*	0.11	-0.346	(-0.6, -0.09)
	D. moj moj	0.05	-0.137	(-0.3, 0.03)
	D. moj baja	0.08	-0.192	(-0.37, -0.01)
V/H FOV ratio	*D. mel*	<.01	-0.004	(-0.17, 0.17)
	D. moj moj	0.01	0.031	(-0.05, 0.12)
	D. moj baja	0.01	-0.052	(-0.18, 0.07)
Ommatidial Count	*D. mel*	0.8**	0.523**	(0.45, 0.59)
	D. moj moj	0.83**	0.653**	(0.57, 0.73)
	D. moj baja	0.74**	0.525**	(0.44, 0.61)
Interommatidial Angle	*D. mel*	0.4**	-0.513**	(-0.68, -0.35)
	D. moj moj	0.49**	-0.467**	(-0.6, -0.34)
	D. moj baja	0.33**	-0.352**	(-0.49, -0.22)
Ommatidial Area	*D. mel*	0.13	0.332	(0.11, 0.56)
	D. moj moj	0.08	0.189	(0.02, 0.36)
	D. moj baja	0.5**	0.509**	(0.37, 0.64)
λ_{max}	*D. mel*	0.11	-0.347	(-0.62, -0.08)
	D. moj moj	0.19	-0.372	(-0.58, -0.16)
	D. moj baja	0.02	-0.098	(-0.29, 0.1)

Table S2: Parameters from the allometric regressions of eye measurements,, related to STAR methods. Asterisks signify the same as in Supplementary Table 1.

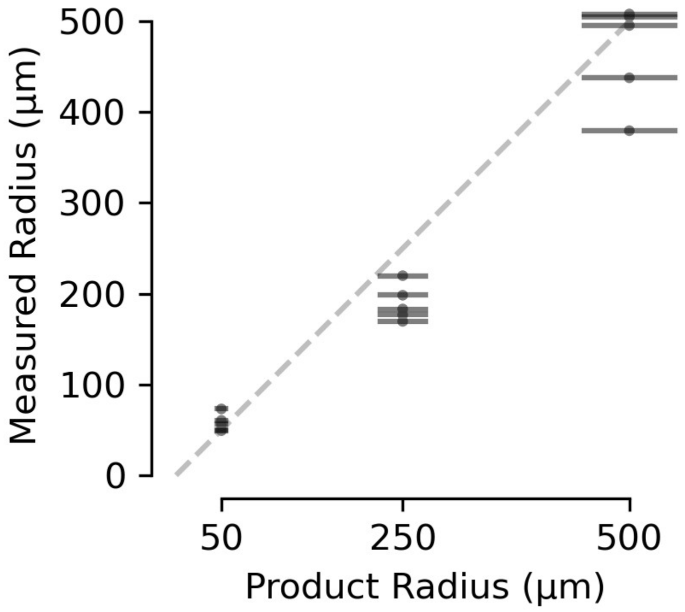

Figure S1: Comparison of diameter measurements produced by our program applied to homogenizing glass beads, related to STAR methods. Beads had radii of 50 ± 5, 250 ± 25, and 500 ± 50 µm, N=5 beads per radius. Dots represent the measured radius (y-axis) for each bead radius according to the product specifications (x-axis). Lines indicate the same as dots except accounting for variability in the product radius (x-axis) according to the product specifications. The dashed gray line indicates the line of equality.

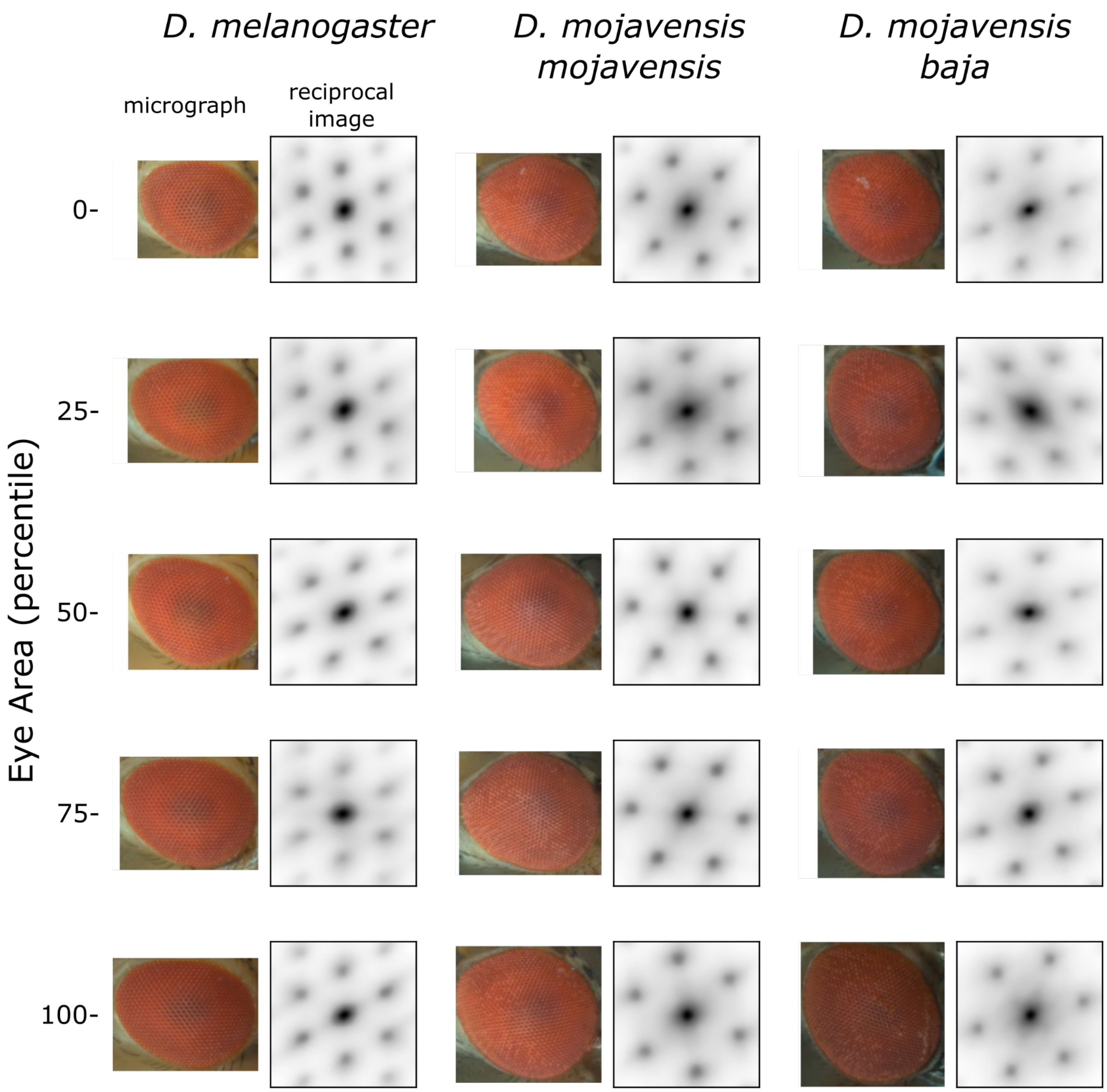

Figure S2: Demonstration of micrographs and their reciprocal image of the 0th (smallest), 25th, 50th, 75th, and 100th (largest) percentile eye of each genotype, related to STAR methods. The reciprocal image is autocorrelated to highlight the recurring maxima representing reciprocal diameters along the major axes of the ommatidial lattice. Notice that *D. mel* eyes are generally smaller than the two desert species, *D. moj moj* and *D. moj baja*. Also, the maxima in the reciprocal images of *D. mel* are closer to the center than the desert species, indicating larger ommatidial diameters in the vinegar flies.

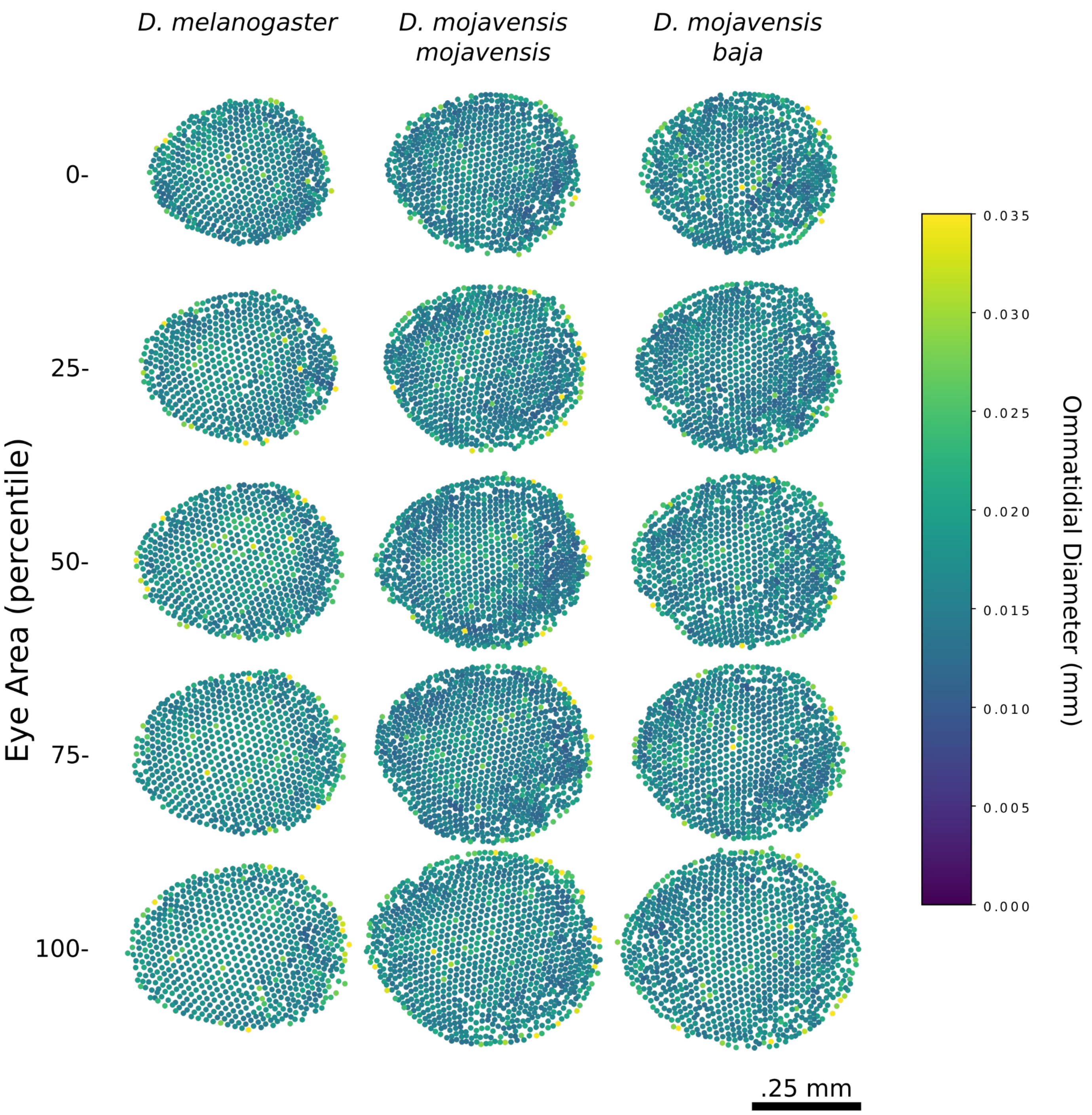

Figure S3: Results of the ODA applied to the eye micrographs in Supplemental Figure 2, related to STAR methods. Each eye is horizontally oriented, dot position indicates the center of an ommatidium, and dot color of each dot indicates its diameter. Notice that *D. mel* eyes have generally larger ommatidia and that there is no dramatic increase in ommatidial density typical of a visual streak for any genotype.
